# Supplementary material for: Influence of nitrogen addition on soil respiration and soil properties in urban forests in Hefei city in China
Source: PLoS One. 2025 Jun 25;20(6):e0324615. doi: 10.1371/journal.pone.0324615 (PMC12192109; doi:10.1371/journal.pone.0324615)
Supplement: S1 File — (DOC) [file pone.0324615.s001.doc]

**The data in Figure 2**

| Measurement time | Experimental sites closer to the suburbs /m(-2)-s(-1) | | | Experimental sites far from the suburbs/m(-2)-s(-1) | | |
| --- | --- | --- | --- | --- | --- | --- |
|  | A | B | C | A | B | C |
| August | 3.32±0.53 | 3.62±0.63 | 3.50±0.31 | 3.21±0.54 | 3.43±0.62 | 3.72±0.62 |
| October | 2.17±0.32 | 2.09±0.41 | 2.15±0.53 | 1.63±0.32 | 1.72±0.41 | 1.62±0.53 |
| December | 0.52±0.16 | 0.48±0.06 | 0.50±0.07 | 0.42±0.21 | 0.37±0.16 | 0.46±0.16 |

**The data in Figure 3**

| Month | Moisture content/% | | | Soil temperature /℃ | | |
| --- | --- | --- | --- | --- | --- | --- |
|  | A | B | C | A | B | C |
| Aug. | 13.22 | 7.91 | 13.63 | 9.32 | 10.02 | 10.12 |
| Sep. | 7.45 | 6.86 | 8.02 | 19.43 | 18.78 | 16.89 |
| Oct. | 5.67 | 4.76 | 5.56 | 22.8 | 21.9 | 19.8 |
| Nov. | 4,62 | 4.57 | 5.09 | 14.5 | 15.09 | 16.89 |
| Dec. | 3.12 | 4.21 | 4.23 | 5.83 | 9.42 | 8.43 |
| Jan. | 1.15 | 1.12 | 2.38 | -8.01 | -7.50 | -7.03 |
| Feb. | 1.56 | 1.55 | 2.47 | -7.32 | -5.32 | -5.32 |
| Mar. | 2.21 | 2.20 | 4.87 | -4.67 | -3.37 | -3.21 |
| Apr. | 2.20 | 2.16 | 4.89 | 4.43 | 4.42 | 4.76 |
| May. | 7.54 | 4.12 | 11.21 | 20.12 | 20.11 | 17.98 |
| Jun. | 11.52 | 6.54 | 10.23 | 20.02 | 18.56 | 20.01 |
| Jul. | 8.65 | 4.98 | 7.68 | 7.67 | 7.12 | 7.12 |

**The data in Figure 4**

| Measure time period | PH value | | |
| --- | --- | --- | --- |
|  | A | B | C |
| August to January | 8.297±0.035 | 8.280±0.022 | 8.165±0.019 |
| February to July | 8.283±0.015 | 8.224±0.020 | 8.218±0.021 |

**The data in Figure 5**

| Measure time period | TN(g/kg) | | |
| --- | --- | --- | --- |
|  | A | B | C |
| August to January | 2.10±0.05 | 2.16±0.14 | 2.12±0.16 |
| February to July | 2.10±0.11 | 2.22±0.18 | 2.21±0.14 |

**The data in Figure 6**

| Measure time period | NO_3_-N(mg/kg) | | |
| --- | --- | --- | --- |
|  | A | B | C |
| August to January | 1.92±0.01 | 3.14±0.07 | 45.78±4.34 |
| February to July | 6.98±0.07 | 8.05±0.02 | 15.11±0.01 |

**The data in Figure 7**

| Measure time period | NH_4_-N(mg/kg) | | |
| --- | --- | --- | --- |
|  | A | B | C |
| August to January | 0.61±0.12 | 1.41±0.21 | 1.12±0.18 |
| February to July | 1.05±0.15 | 1.15±0.41 | 1.20±0.08 |

**The data in Figure 8**

| Soil heterotrophic respiration rate/μMol.m^-2^.s^-1^ | Experimental sites closer to the suburbs | | | Experimental sites far from the suburbs | | |
| --- | --- | --- | --- | --- | --- | --- |
|  | A | B | C | A | B | C |
| August | 1.15±0.13 | 1.28±0.12 | 1.22±0.01 | 0.88±0.21 | 0.94±0.04 | 1.04±0.04 |
| October | 0.73±0.02 | 0.71±0.03 | 0.73±0.01 | 0.52±0.02 | 0.55±0.01 | 0.51±0.03 |
| December | 0.24±0.01 | 0.19±0.02 | 0.23±0.01 | 0.13±0.01 | 0.12±0.02 | 0.1**4**±0.02 |

**The data in Figure 9**

| P | PH | N | RH | RS | RA | TN |
| --- | --- | --- | --- | --- | --- | --- |
| PH | \ | \ | \ | \ | 0.53* | \ |
| N | \ | \ | \ | \ | \ | 0.44* |
| RH | \ | \ | \ | 0.42* | 0.54* | 0.41* |
| RS | \ | \ | 0.42* | \ | 0.44* | \ |
| RA | 0.53* | \ | 0.54* | 0.44* | \ | \ |
| TN | \ | 0.44* | 0.41* | \ | \ | \ |
