# Supplementary material for: Influence of nitrogen addition on soil respiration and soil properties in urban forests in Hefei city in China
Source: PLoS One. 2025 Jun 25;20(6):e0324615. doi: 10.1371/journal.pone.0324615 (PMC12192109; doi:10.1371/journal.pone.0324615)
Supplement: Attachment — Submitted filename: PONE-D-24-51919_reviewer.pdf [file pone.0324615.s002.pdf]

Manuscript Click here to access/download;Manuscript;manuscript.doc

Influence of nitrogen addition on soil respiration and soil properties in urban forests in Hefei city in China

Yueqin Chen and Honghua Ruan*

College of Biology and the Environment, Co-Innovation Center for Sustainable Forestry in Southern China, Nanjing Forestry University, Nanjing 210037, China

***Correspondence: author. Email: crescentmoon1631@163.com

Abstract: With the continuous development of urbanization, nitrogen deposition has affected the regeneration capacity of urban forest soil. This study focused on *Quercus acutissima* Carruth forest in a southern city Hefei in China. The effects of different nitrogen concentrations on soil respiration rate, soil properties of urban forest were studied. In addition, the effects of seasonal changes on soil respiration were also analyzed. The experimental results showed that low-concentration nitrogen (50 g N·m^{-1}) increased soil respiration rate by about 11% in summer, but decreased by about 6% in winter, with a range of 5% to 20%, and the results were significantly different (p<0.05). The addition of high concentration nitrogen (100 g N·m^{-1}) significantly increased soil temperature (about 12%) in summer and decreased it by about 13% in winter (p<0.05), which revealed the seasonal regulation effect of nitrogen concentration on soil temperature. Soil moisture content and temperature have significant effects on soil respiration rate, especially in the suburban test site, the correlation coefficient R^2 is close to 1. Nitrogen addition significantly increased soil total nitrogen and nitrate nitrogen contents (p<0.05), but had no significant effect on soil pH value. Structural equation model analysis showed that nitrogen addition directly or indirectly regulated soil heterotrophic respiration and autotrophic respiration by affecting soil nutrient status. The findings not only deepen the understanding of the ecological effects of nitrogen deposition, but also provide an important scientific basis for the assessment and management of soil carbon cycling in the process of urbanization. In conclusion, this study revealed the significant effects of nitrogen addition on urban forest soil respiration and its components, which provided data support and theoretical reference for understanding the dynamic changes of urban ecosystem carbon cycle, and was of great significance for guiding future urban greening and ecological protection work.

Keywords: Soil properties; Forest soil; *Quercus acutissima* Carruth

1. INTRODUCTION

When socio-economic develops rapidly, the expansion of human activities leads to a sharp increase in global nitrogen deposition over the past century. The nitrogen input in terrestrial ecosystems increases nearly fivefold, having a significant impact on the global nitrogen cycle [1]. Excessive nitrogen input may lead to soil acidification and biodiversity decline [2-3].

Soil respiration is the process by which carbon dioxide (CO_2) is released from the soil, and soil respiration releases about ten times as much CO_2 as fossil fuel emissions [4]. CO_2 is released mainly through the respiration of microorganisms and plant roots, which is a key link in the global carbon cycle and plays an important role in the terrestrial ecosystem [5-6]. Soil respiration is mainly affected by soil properties, soil nutrients, pH value, temperature and moisture. The properties of soil, such as soil structure, texture, and organic matter content, directly affect the activity of soil microorganisms and roots, thereby regulating soil respiration rate. The soil nutrients, such as nitrogen, phosphorus, and potassium, significantly affect the growth and metabolic activities of microorganisms, thereby affecting soil respiration. Different microorganisms have different adaptation ranges to pH values. Environments that are too acidic or too alkaline can limit the activity of certain microorganisms, thereby affecting soil respiration. Temperature and humidity are two important environmental factors affecting soil respiration, which regulate soil respiration rate mainly by affecting microbial activities and plant root activities [7]. Usually, an increase in temperature promotes soil respiration. The moisture content in soil mainly affects the diffusion of oxygen and the activity of microorganisms.

The contents of nitrogen in the soil will increase as nitrogen deposition increases, which may significantly affect the characteristics of forest soil and thus affect soil respiration[8]. Nitrogen Addition (NA) refers to the process in which nitrogen elements in the atmosphere settle into the soil. At present, some studies believe that NA can promote soil respiration and thus reduce soil carbon content [9]. However, there are also studies suggest that NA may increase carbon gain generated by photosynthesis of terrestrial plants, thereby increasing soil carbon content [10]. In fact, the effects of NA on soil respiration are mainly realized through changes in plant root activities, microbial biomass, and soil carbon content [11]. Similar studies on the impact of urban forest soil respiration and its composition in this article are very rare. Some researchers believe that NA and soil

physicochemical properties have a significant impact on soil respiration [12]. Han Y analyzed the effect of NA on changes in arbuscular mycorrhizal communities [13], and found that NA significantly reduced the overall abundance of arbuscular mycorrhizal fungi, thereby affecting the response of root biomass changes and ultimately affecting soil pH. The results indicated that NA indirectly reduces soil respiration rate by reducing the abundance of arbuscular mycorrhizal fungi and altering root biomass. Hu J et al. compared 100 microbial data pairs using meta-analysis methods to study the carbon utilization efficiency of microorganisms [14]. Various methods for estimating carbon were used to analyze the response information of microbial carbon utilization efficiency after NA. The results showed that NA reduced microbial carbon utilization by 12%, indicating that NA affected microbial metabolism. Li Xet al. conducted a field experiment using temperate deciduous forests to investigate the effects of different NA on the dynamics of host roots in temperate forests [15]. The dynamic changes of host roots in the lower canopy and under the forest with different NA were compared. The results showed that higher root yield under low canopy will lead to higher soil respiration rate [16]. Molaei A M et al. conducted comparative experiments based on four different soil management methods to analyze the impact of land use efficiency on soil properties. The research results of Molaei AM indicate that land use changes from natural forests to other uses have a significant impact on most research parameters. Different land use methods can affect the percentage of particle size distribution, Therefore, the results showed that natural forests and walnut orchards have higher organic matter content and better water retention ability due to their higher clay ratio. These factors can promote soil microbial activity and root respiration, thereby increasing soil respiration rate [17]. A total of 40 sample data were collected and their soil characteristics were measured. The results showed that poor land management led to an increase in soil pH value. Land use had a significant impact on the content of calcium carbonate in soil. Therefore, land use change had a significant impact on soil properties. Especially in China, research on NA and urban forest soil respiration is still in its early stages [18]. Currently, most research on soil respiration focuses on agricultural systems or natural forests, while urban forests, as a special environment, are affected by human activities and high nitrogen deposition, and the response mechanisms of their soil respiration and its components have not been fully studied. In order to fill this research gap and reveal the ecological process of

urban forest soil under NA conditions, this study analyzes the impact of NA on soil respiration and its components, in order to reveal its mechanism of action and provide scientific basis and reference for optimizing urban forest management strategies.

In summary, research on how NA and physicochemical properties affect soil respiration has been discussed by several researchers, and most of these studies are focused on agricultural systems or natural fields. However, there are significant differences between urban forests and natural forests. For example, urban forests are often affected by more human activities, higher heavy metal pollution, higher soil compaction, and different water and temperature conditions, all of which can affect soil respiration. In addition, nitrogen deposition in urban forests is usually high, originating from nitrogen oxides emitted from transportation, industry, and residential areas. These factors can lead to differences in nitrogen cycling in urban forest soils compared to natural forests. However, there is currently relatively little research on soil respiration in urban forests, especially on the effects of NA. Therefore, in order to fill the research gap in this field, this study innovatively investigates the impact of NA in urban forests on soil respiration, revealing soil ecological processes and mechanisms different from natural forests, in order to enrich the theoretical system of soil ecology.

2. MATERIALS AND METHODS

2.1. Overview of the research site

Quercus acutissima Carruth forest is selected as the experimental object in this study [19]. This forest is located in the suburbs of Hefei city in China. According to the distance from the city center, the experimental area is divided into exurb experimental area and suburban experimental area. The experimental area belongs to a subtropical monsoon climate, with an average annual precipitation of about 1000 millimeters. The climate conditions are humid and mild, with an average relative moisture of 79%. The average annual temperature in the region is maintained at around 17 ℃, providing an ideal environment for the growth of *Quercus acutissima* Carruth. The exurb experimental area is located in an area at an altitude of 142 meters, with a local NA level detection value of 16.43 kilograms per hectare. The distribution density of *Quercus acutissima* forest in this experimental area is 837 plants per hectare, with a canopy density of 0.75. The average diameter at breast height of *Quercus acutissima* is 33.54 centimeters. The average tree height is 19.75 meters.

The suburban experimental area is located at an altitude of 92 meters, with a NA level of 18.52 kilograms per hectare. The distribution density of *Quercus acutissima* forest is slightly low, with 813 plants per hectare, a canopy density of 0.70, an average diameter at breast height of 25.62 centimeters, and an average height of 16.48 meters. The above data are obtained through on-site investigation and measurement. The average breast diameter of *Quercus acutissima* trees is measured using a tape measure at the height of the tree trunk, which is 1.3 meters above the ground. The height of the *Quercus acutissima* tree is obtained using a height gauge. Density and canopy closure are calculated by counting the number of trees in the sample plot to determine the distribution density between forests. Canopy closure is evaluated through canopy coverage. The map of the research area is shown in Figure 1.

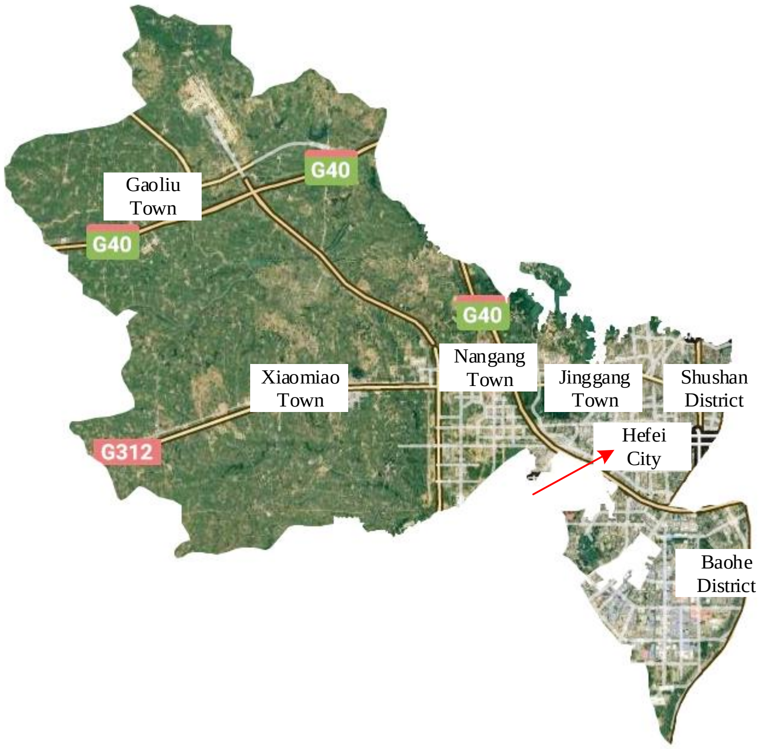

Figure 1. Research area map.

2.2. Research methods

2.2.1. Experimental area design

The selection of the *Quercus acutissima* Carruth. Forest experimental area should meet the following requirements. Firstly, the area of the experimental area must be greater than 55 m × 55 m. A buffer zone of approximately 10m wide is set around the experimental area to ensure the independence of the experiment and reduce external interference. Secondly, the experimental area

should have a rich variety of species, a balanced ecological environment, and a complete community structure to ensure the ecological representativeness of the experiment. In addition, the selection of experimental areas should avoid complex terrains such as trench bottoms or mountain tops. It is necessary to select areas that are relatively flat or uniformly gentle to carry out and manage experiments. Each experimental area is evenly divided into 9 plots, each of which is a 15 m × 15 m square with an area error controlled within 0.5 m. The plots are numbered sequentially from 1 to 9. Figure 2 (a) is a schematic diagram of the experimental area design. In Figure 2 (a), starting from 2022, nitrogen deposition simulation experiments are designed and planned in the experimental area, and three different concentrations of nitrogen deposition are simulated on 9 plots in the experimental area. Zone A is the control area, and no treatment or fertilization is done during the non-growing period. Zone B is applied with low nitrogen, and the amount of nitrogen applied per square meter is 50 g/year, that is, 50 g $N \cdot m^{-2} \cdot yr^{-1}$. High nitrogen is applied in Zone C at 100 g per square meter per year, that is, 100 g $N \cdot m^{-2} \cdot yr^{-1}$. The simulated nitrogen deposition fertilizer is ammonium nitrate (NH_4NO_3). During the plant growth season, Zones B and C are fertilized every two months [20]. In addition, in order to separate heterotrophic respiration (RH) and autotrophic respiration (RA), the physical separation method was used. As shown in Figure 2 (b), the study set up three soil rings in each experimental plot, of which two rings were used to measure total soil respiration (RS) and the other ring was used to measure heterotrophic respiration (RH) of rootless soil by digging trenches to remove roots. By calculating the difference between RS and RH, the rate of autotrophic respiration (RA) can be obtained. The Soil Master Kit portable soil CO_2 rapid tester (Spectrum, USA) is used to measure the CO_2 content in the soil.

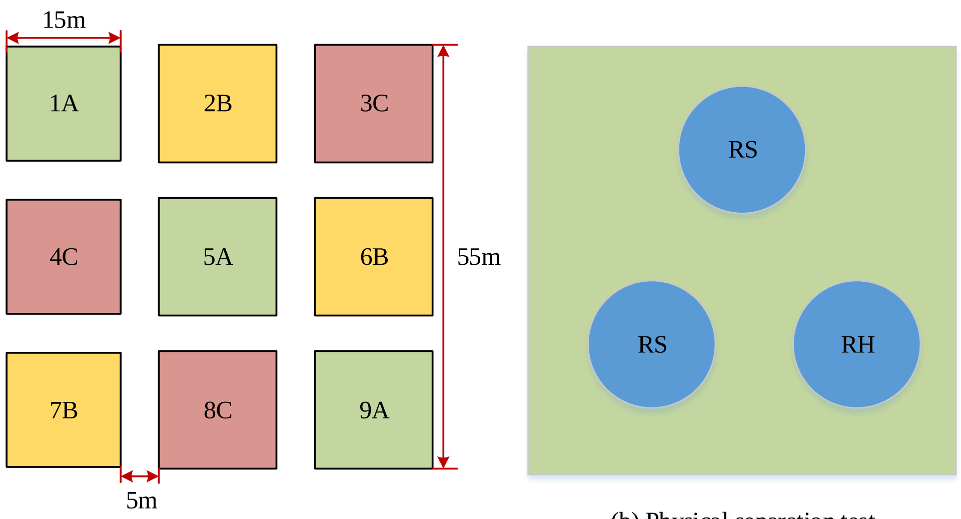

(a) Experimental site design

(b) Physical separation test partition

Figure 2. Schematic diagram of experimental area design.

2.2.2. Collection of experimental data

Soil samples are collected using a soil drill. Three sampling points are randomly selected within the sample plot. Soil samples are taken from three soil layers of 0-20 cm, 20-40 cm, and 40-60 cm, respectively. A 2mm sieve is used for sampling after mixing the soil from the same soil layer at the three sampling points. In this study, soil samples collected from the experimental area are quickly sealed and stored in an environment of approximately 4°C to maintain microbial activity [21-22]. Pretreatment of these samples includes filtration in solution and drying after filtration. A funnel and filter paper are used to filter out solid particles and impurities in the soil during solution filtration. Firstly, the filter paper is moistened with a small amount of distilled water, then the solution is slowly poured into a funnel to pass through the filter paper. These samples are placed in the experimental oven for thorough drying after air drying. Finally, these samples are screened for soil particles through a 70 mesh sieve. The drying temperature of the soil is between 105°C and 110°C. The drying time of the soil is 24 hours. The screened soil samples are thoroughly mixed, labeled, and encapsulated for subsequent analysis. Filtration by solution is studied to remove impurities and soluble salts from the soil in order to determine soil chemistry more accurately. It is true that some soluble nitrogen and other dissolved substances may be lost during filtration, but this loss is a small proportion of the total nitrogen addition, and this has been taken into account in the analysis. At the same time, chemical analysis of the filtered filtrate was also carried out to ensure the

accuracy and reliability of the data. After soil treatment, the following soil variables will be measured, such as soil temperature, soil pH value, soil moisture content, and chemical properties such as nitrogen in the soil.

2.3. Soil respiration measurements

An in-depth understanding of soil properties is particularly critical when conducting soil respiration research. The study focused on the hemp oak forest on the outskirts of a southern city. The soil type in this area is subtropical forest soil rich in organic matter, which has good water retention and air permeability, and supports the luxuriant growth of the hemp oak tree and the vegetation under the forest. The soil texture is mainly loam or clay loam, with a relatively high proportion of silt particles, supplemented by an appropriate amount of clay and sand particles, which helps to maintain the stability of soil structure and the sustainable supply of nutrients, creating an ideal living environment for microorganisms and plant roots. According to the distance from the urban center, the study area was divided into outer suburbs and outer suburbs. Although the soil types of the two sites were similar, they showed significant differences in soil thickness, nutrient content, microbial community structure and pH value. Specifically, surface soil (0-20cm), as the main layer of soil respiration, is rich in organic matter and active in biological activities. Due to the influence of more human activities (such as fertilizer application and garbage disposal), the soil nutrient content is higher and the microbial community is more complex and diverse. The exurbs remained relatively natural, and the microbial communities were dominated by saprophytic bacteria and rhizosphere microorganisms. The natural difference of soil pH value is also affected by many factors such as soil parent material, climatic conditions and vegetation types. Soil respiration is measured using chamber method. Three soil rings are set up in each experimental area, with each ring spaced approximately 3 meters apart [23]. The area of the soil ring is approximately 0.0318 square meters. The height of the soil collars is 9cm, with 4cm buried in the soil and the rest exposed to the surface [24]. A respiration apparatus (Li-8100 produced by Li Cor in the United States) is used to measure soil respiration on a soil ring. The measured respiration heterotrophic (RH) of the soil is subtracted from the soil environmental measurement value to accurately determine the root respiration value of the soil. The difference obtained is the root respiration value of the soil [25]. The method for measuring RH is the trench method, which

involves setting three measuring points approximately 2.5 m from the edge of the sample plot. Each measured area is approximately 1 m × 1 m. A trench of about 1m is dug around the measuring point. The soil is backfilled to a level after dividing the tree roots with plastic cloth [26]. The depth of the root of trees in the soil (5-10 cm) is usually chosen as the measurement depth to better reflect the temperature state of the root active zone and thus determine the effect of temperature on soil respiration. Since soil respiration rate varies significantly throughout the day and is affected by factors such as temperature, humidity, and plant photosynthesis, different time periods of the day are selected to measure SR and ST. By measuring multiple times during the 8 a.m. - 6 p.m. period, the daily variation characteristics of soil respiration can be captured, providing more comprehensive data. Before measurement, it is necessary to remove garbage and shrubs near the experimental area to eliminate interference from other factors [27-28]. The initial time for soil respiration measurement is set in August 2022. After that, the experiment was conducted every two months for a total of three times. Each measurement lasted about 100 minutes, from 8 a.m. to 6 p.m., with an average of 20 minutes measured every two hours. In order to ensure the accuracy and consistency of the data, soil moisture and soil respiration were measured at the same time period. In addition, soil temperature is measured monthly from August 2022 to July 2023 to collect soil temperature data throughout the year. The repeated measurements at each site are three times a month, with each measurement sampled from three different soil environments and repeated to ensure the accuracy of the data. The content of soil moisture is determined by the drying method. Firstly, the fresh soil brought back to the laboratory is poured into a clean aluminum box. The total weight of the fresh soil and aluminum box is weighed. The soil is then placed in an oven set at 105℃ and dried to a constant weight. The total weight of the dry soil and aluminum box is weighed. Finally, the aluminum box is separately weighed to determine the soil moisture content.

2.4. Determination of soil chemical properties

The soil moisture was measured by drying method. First, the fresh soil is poured into a clean aluminum box after being brought back to the laboratory, and the total weight of the fresh soil and aluminum box is weighed. Then, the soil is dried in the oven at 105 °C to constant weight, and the total weight of the dry soil and the aluminum box is weighed again. By calculating the quality

difference between fresh soil and dry soil, the moisture content of soil can be obtained. Flow injection apparatus and multi-N/C instrument are used to determine chemical properties such as nitrogen. The flow injection instrument mainly measures the content of nitrate nitrogen and ammonium nitrogen in soil. The multi-N/C instrument measures soluble organic carbon in soil [29-30]. The content of available phosphorus was determined using the Olsen method. In addition, the pH value of the soil will be determined using potentiometric analysis. The analysis of total soil nitrogen is determined using a 3000 element analyzer. The determination of electrical conductivity is carried out on soil solutions using a conductivity meter. Statistical analysis is conducted by using software such as Excel, SPSS, and MATLAB. First, the collected statistical testing data are organized, using Excel software for data entry, preliminary organization, and basic descriptive statistical analysis. Then, SPSS software is used to perform correlation analysis, regression analysis, and analysis of variance on the data. Finally, the MATLAB software is used to model and visualize the data.

2.5 Data analysis methods

In this study, Excel, SPSS, MATLAB and other software were used for statistical analysis. After data collection, Excel was used for preliminary data collation and descriptive statistical analysis. Then, SPSS software was used for correlation analysis, regression analysis and analysis of variance (ANOVA). In order to verify the significant effects of different nitrogen addition treatments on soil respiration rate and other parameters, one-way analysis of variance (ANOVA) was performed, and LSD (minimum significant difference method) was used for multiple comparisons to determine whether the differences among treatments were statistically significant (p<0.05). Prior to ANOVA, the data were tested for normality and homogeneity of variance. The data satisfy the assumptions of normal distribution and homogeneity of variance, so the ANOVA results are reliable. The specific statistical indicators include F-value, p-value, mean difference and confidence interval between different treatments. Finally, MATLAB software is used to model and visualize the data.

3. RESULT ANALYSIS

3.1. Influence of NA on soil respiration in urban forests

3.1.1. Influence of NA on soil respiration rate

Figure 3 shows how NA affects soil respiration rate. From August to December, the soil respiration rate in both the suburban and exurb experimental areas showed a decreasing trend (p<0.05). As can be seen from Figure 3 (a), in the suburban experimental area, compared with the soil respiration rate at nitrogen deposition concentration A, the soil respiration rate at nitrogen deposition concentration B and C all increased in August. But in October, there was a decline. The soil respiration rates of nitrogen deposition concentration B and concentration C in August were 3.62±0.63 μmol·m^{-2}·s^{-1} and 3.50±0.31 μmol·m^{-2}·s^{-1}. The soil respiration rate increased by 11.04% and 7.36% compared with the soil respiration rate of 3.26±0.45 μmol·m^{-2}·s^{-1} at concentration A at this time (p<0.05). The soil respiration rates of nitrogen deposition concentration B and concentration C in December were 0.48±0.06 μmol·m^{-2}·s^{-1} and 0.50±0.07 μmol·m^{-2}·s^{-1}. The soil respiration rate decreased by 5.88% and 1.96% compared with the soil respiration rate of 0.51±0.07 μmol·m^{-2}·s^{-1} at concentration A at this time (p<0.05). The addition of low-concentration nitrogen increased soil respiration rate by about 5% to 20%, and there was a significant difference in the results (p<0.05). Low concentration nitrogen promoted soil respiration, while high concentration nitrogen inhibited soil respiration, with changes of around 5%. The results showed significant differences (p<0.05).

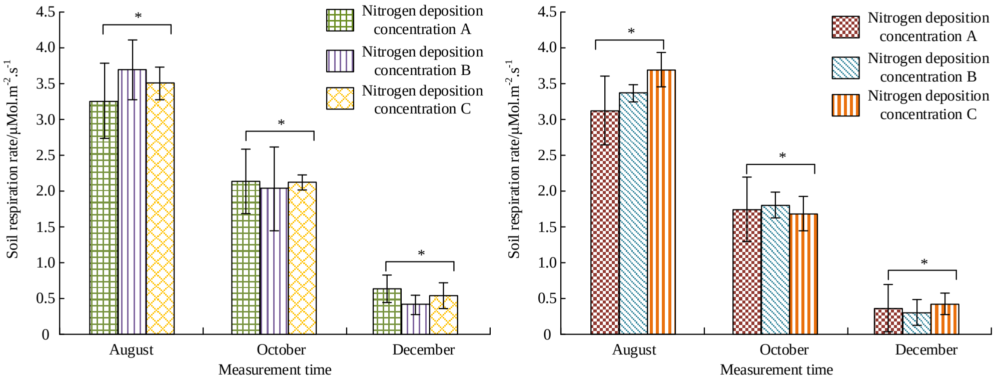

(a) Experimental sites closer to the suburbs (b) Experimental sites far from the suburbs

Figure 3. Influence of NA on soil respiration rate(*Indicating p<0.05).

3.1.2. Influence of NA on soil moisture and temperature

Figure 4. shows how NA affects soil moisture content and temperature in urban forests. In Figure 4 (a), the moisture content of forest soil fluctuated between 1.12% and 13.72%, reaching the lowest and highest values in January and August, respectively. In January, the moisture content of nitrogen deposition concentrations A, B, and C were 1.15%, 1.12%, and 2.38%, respectively. In

January, compared with the control group A, B decreased by only 0.03%, basically unchanged, while C increased by 1.23%. In August, the moisture content of nitrogen deposition concentrations A, B, and C were 13.22%, 7.91%, and 13.63%, respectively. Compared with control group A, B decreased by 5.31% and C increased by 0.41%. Therefore, low-nitrogen reduced soil moisture content, while high-nitrogen didn't affect soil moisture content obviously. The reason for this result may be that low nitrogen treatment promotes plant growth and increases the absorption and utilization of water by plants, resulting in a relative reduction of water content in the soil. However, this reduction may not be statistically significant and is influenced by a variety of environmental factors. In Figure 4 (b), the soil temperature reached the lowest and highest values in January and October, respectively, with temperature fluctuations ranging from -8.01 ℃ to 22.57 ℃. In January, the temperature of nitrogen deposition concentrations A, B, and C were -8.01 ℃, -7.50 ℃, and -7.03 ℃, respectively. Compared with control group A, the temperature increased by 6.36% and 12.23%, respectively. In October, the temperature of nitrogen deposition concentrations A, B, and C were 22.8 ℃, 21.9 ℃, and 19.8 ℃, respectively. Compared with control group A, the temperature decreased by 3.94% and 13.15%, respectively. The above results show that low nitrogen has no significant effect on soil temperature, while high nitrogen increases and decreases soil temperature. The reason for this result may be that nitrogen addition indirectly affects soil temperature by changing the physical properties of soil and the decomposition rate of organic matter. For example, nitrogen addition may promote soil microbial activity and release heat, thereby increasing soil temperature to some extent. However, this effect is limited by a variety of factors, such as soil moisture and vegetation cover. In summary, it can be seen that low nitrogen concentration mainly affects soil moisture content by promoting plant growth and increasing water absorption, while high nitrogen concentration affects soil temperature by changing soil properties and organic matter decomposition rate.

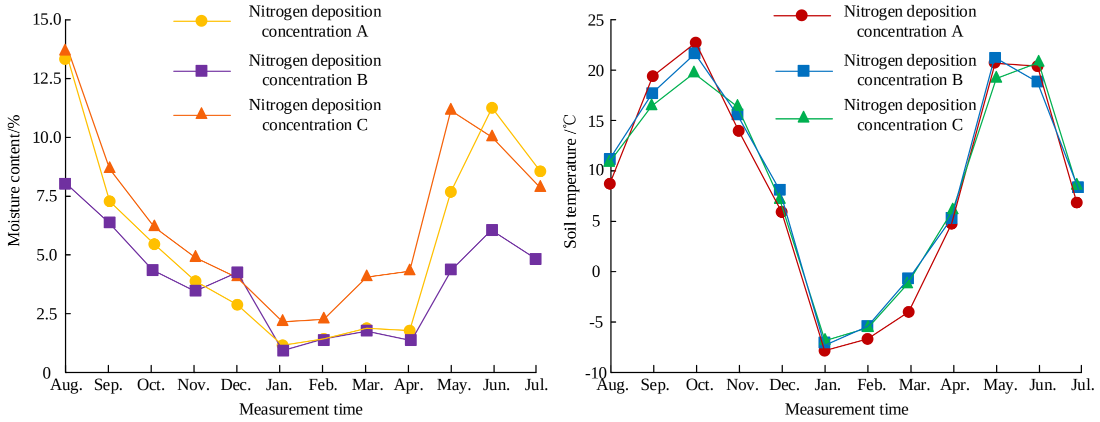

(a) Monthly average moisture content of urban forest soil under nitrogen addition

(b) Monthly average temperature of urban forest soil under nitrogen addition

Figure 4. Influence of NA on soil moisture content and temperature in urban forests.

3.1.3. Correlation analysis between soil respiration rate and soil moisture

Table 1 presents the correlation between soil respiration rate and soil moisture. When the nitrogen deposition concentration was 0, R^2, Q_{10}, and p were 0.993, 2.05, and 0.042, respectively, indicating that soil respiration rate and water content had an obvious correlation ($p<0.05$). When the nitrogen deposition concentrations were 50 g $N \cdot m^{-2} \cdot yr^{-1}$ and 100 g $N \cdot m^{-2} \cdot yr^{-1}$, the R^2 slightly decreased compared to the nitrogen deposition concentration 0. However, Q_{10} values of 2.11 and 2.00 were relatively high, indicating that moisture content still affected soil respiration ($p<0.05$). The R^2 and Q_{10} with different concentrations of nitrogen added in the exurb experimental area were lower than those in the suburban experimental area. Therefore, the correlation between soil respiration rate and soil moisture content in suburban experimental areas was relatively low. This is because NA can alter the nutrient conditions in the soil, thereby affecting characteristics such as soil temperature and moisture.

Table 1. Correlation between the soil respiration rate and soil moisture (*Indicating $p<0.05$).

Experimental area	Nitrogen deposition concentration	Factor 1	Factor 2	Parameters		
				R^2	Q_{10}	p-value
Experimental areas closer to the suburbs	A	0.068	0.281	0.993	2.05	0.042*
	B	0.041	0.319	0.959	2.11	0.026*
	C	0.074	0.266	0.956	2.00	0.031*
Experimental areas far from the suburbs	A	0.069	0.140	0.632	0.97	0.004*
	B	0.085	0.127	0.559	0.86	0.016*

	C	0.052	0.157	0.700	0.18	0.044*

3.1.4. Correlation analysis between soil respiration rate and temperature

The respiration rate of forest soil is closely related to soil temperature. The correlation between the two was evaluated to understand the impact of soil temperature on soil respiration rate in Table 2. The R^2 and Q_{10} of the suburban experimental area were generally higher than those of the exurb experimental area. This indicated that soil respiration rate and soil temperature's correlation in the suburban experimental area was greater than that in the exurb experimental area. When nitrogen was added to concentration A in the suburban experimental area, the R^2 and Q_{10} were 0.786 and 2.05, respectively. This indicated that soil respiration rate and soil temperature had a positive correlation obviously (p<0.05). When nitrogen was added at concentration B, the R^2 and Q_{10} were 0.876 and 2.11, respectively, which were 11.45% and 2.92% higher than when nitrogen was added at concentration A. The increase in nitrogen deposition concentration enhanced soil respiration's sensitivity to soil temperature. From the exurb experimental area, the R^2 was generally low, indicating a weak correlation between soil respiration rate and soil temperature. When nitrogen was added at concentration A, although the p-value was 0.024, the R^2 and Q_{10} were 0.769 and 0.97, respectively, indicating lower values.

Table 2. Correlation between the soil respiration rate and temperature (*Indicating p<0.05).

Experimental area	Nitrogen deposition concentration	Intercept	Slope	Parameters		
				R^2	Q_{10}	p-value
Experimental areas closer to the suburbs	A	0.223	-1.545	0.786	2.05	0.039*
	B	0.283	-2.490	0.876	2.11	0.008*
	C	0.210	-1.510	0.783	2.00	0.029*
Experimental areas far from the suburbs	A	0.147	-1.373	0.769	0.97	0.024*
	B	0.117	-0.894	0.633	0.86	0.036*
	C	0.178	-1.866	0.883	0.18	0.049*

3.2. Influence of NA on soil properties

3.2.1. Influence of NA on soil pH value

Figure 5 shows the effect of NA on the soil pH. From August to January, the pH values of nitrogen deposition concentrations 0, 50 g $N \cdot m^{-2} \cdot yr^{-1}$, and 100 g $N \cdot m^{-2} \cdot yr^{-1}$ were 8.297±0.035,

8.280±0.022, and 8.165±0.019, respectively. Compared with concentration 0, the pH of 50 g $N \cdot m^{-2} \cdot yr^{-1}$, and 100 g $N \cdot m^{-2} \cdot yr^{-1}$ decreased by 0.20% and 1.59%, respectively. From February to July, the pH values of nitrogen deposition concentrations 0, 50 g $N \cdot m^{-2} \cdot yr^{-1}$, and 100 g $N \cdot m^{-2} \cdot yr^{-1}$ were 8.283±0.015, 8.224±0.020, and 8.218±0.021, respectively. Compared with concentration 0, the pH of 50 g $N \cdot m^{-2} \cdot yr^{-1}$, and 100 g $N \cdot m^{-2} \cdot yr^{-1}$ decreased by 0.71% and 0.78%, respectively. Therefore, the pH of the soil treated with low and high-nitrogen showed a slight decrease compared to the control group with nitrogen blank, but not significant (p>0.05).

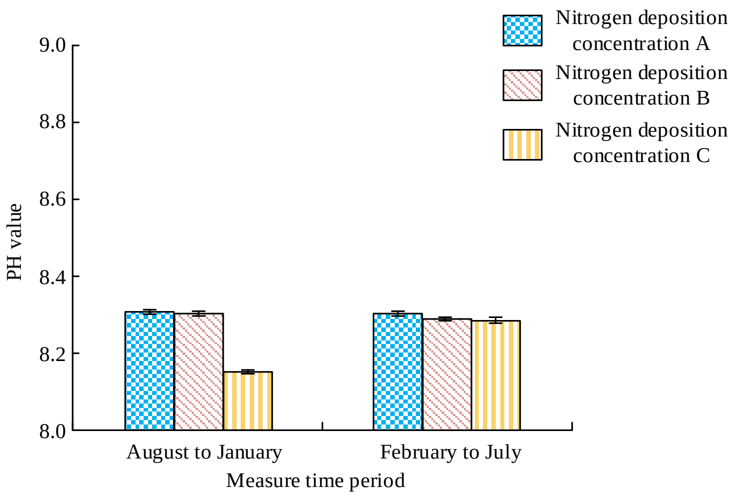

Figure 5. Influence of NA on soil pH value(*Indicating p<0.05).

3.2.2. Influence of NA on total soil nitrogen

Figure 6 shows how NA affects total soil nitrogen. During a period from August to January, the total nitrogen of nitrogen deposition concentrations 0, 50 g $N \cdot m^{-2} \cdot yr^{-1}$, and 100 g $N \cdot m^{-2} \cdot yr^{-1}$ was 2.10±0.05 g/kg, 2.16±0.14 g/kg and 2.12±0.16 g/kg, respectively. Compared with concentration 0, the total nitrogen of 50g $N \cdot m^{-2} \cdot yr^{-1}$, and 100g $N \cdot m^{-2} \cdot yr^{-1}$ increased by 2.85% and 0.95%, respectively. From February to July, the total nitrogen of nitrogen deposition concentrations 0, 50 g $N \cdot m^{-2} \cdot yr^{-1}$, and 100 g $N \cdot m^{-2} \cdot yr^{-1}$ was 2.10±0.11 g/kg, 2.22±0.18 g/kg, and 2.21±0.14 g/kg, respectively. Compared with concentration 0, the total nitrogen of 50 g $N \cdot m^{-2} \cdot yr^{-1}$, and 100 g $N \cdot m^{-2} \cdot yr^{-1}$ increased by 5.71% and 5.23%, respectively. Therefore, the total nitrogen of the soil treated with low and high-nitrogen showed a slight increase compared to the total nitrogen content of the control group soil with nitrogen blank (p<0.05).

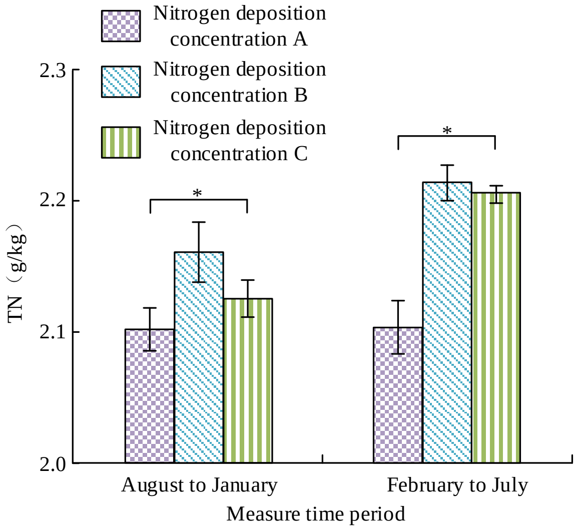

Figure 6. Influence of NA on total soil nitrogen(*Indicating $p<0.05$).

3.2.3. Influence of NA on soil nitrate nitrogen

During a period from August to January, the nitrate nitrogen deposition concentrations A, B, and C were 1.92±0.01 mg/kg, 3.14±0.07 mg/kgand 45.78±4.34 mg/kg, respectively. Compared with concentration A, the nitrate nitrogen of B increased by 1.22 mg/kg, and the nitrate nitrogen of C increased by 43.86 mg/kg. From February to July, the nitrate nitrogen deposition concentrations A, B, and C were 6.98±0.07 mg/kg, 8.05±0.02 mg/kg, and 15.11±0.01 mg/kg, respectively. Compared with concentration A, the nitrate nitrogen of B increased by 1.07 mg/kg, and the nitrate nitrogen of C increased by 8.13 mg/kg.Therefore, the soil nitrate nitrogen in the low-nitrogen and high-nitrogen treatments increased obviously compared to the nitrogen blank control group ($p<0.05$).

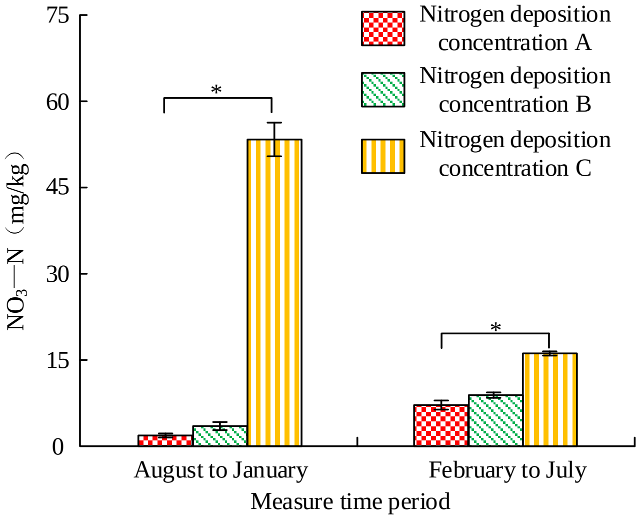

Figure 7. Influence of NA on soil nitrate nitrogen(*Indicating p<0.05).

3.2.4. Influence of NA on soil ammonium nitrogen

Figure 8 shows how NA affects soil ammonium nitrogen. From August to January, the ammonium nitrogen deposition concentrations A, B, and C were 0.61±0.12 mg/kg, 1.41±0.21 mg/kg and 1.12±0.18 mg/kg, respectively. Compared with concentration A, the ammonium nitrogen of B increased by 0.80 mg/kg, and the nitrate nitrogen of C increased by 0.51 mg/kg. From February to July, the ammonium nitrogen deposition concentrations A, B, and C were 1.05±0.15 mg/kg, 1.15±0.41 mg/kg, and 1.20±0.08 mg/kg, respectively. Compared with concentration A, the ammonium nitrogen of B increased by 0.10 mg/kg, and the nitrate nitrogen of C increased by 0.15 mg/kg.Therefore, the ammonium nitrogen in the soil treated with low and high-nitrogen showed a significant increase compared to the control group soil with nitrogen blank from August to January. There was a slight increase from February to July (p<0.05).

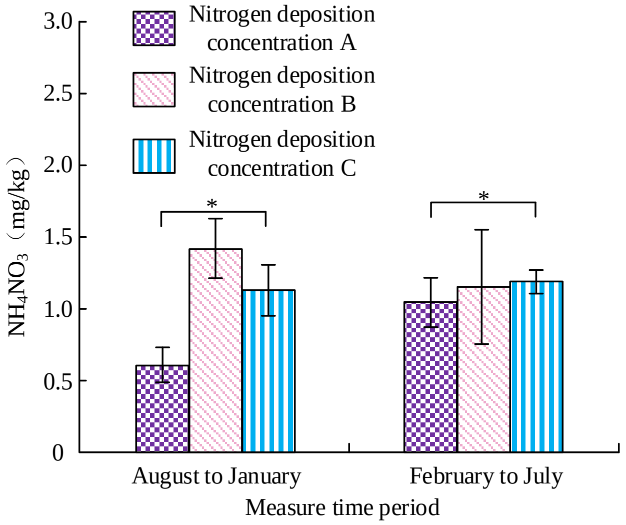

Figure 8. Influence of NA on soil ammonium nitrogen(*Indicating p<0.05).

3.2.5. Correlation analysis of NA on soil properties

Table 3 shows different NAs and soil properties' correlation. The chemical properties of soil, including NA, nitrate nitrogen, and ammonium nitrogen, showed significant changes under the comprehensive effects of treatment, time, and experimental area effects (p<0.05). PH was not related to any influencing factors (p>0.05). The total nitrogen content was significantly affected by time, experimental area effect, treatment and experimental area interaction, time, and experimental area interaction factors (p<0.05). The p-value of the carbon to nitrogen ratio under the experimental

area effect was 0.043. The *p*-value under the interaction of time and experimental area was 0.049. Therefore, the carbon to nitrogen ratio showed significant differences under the influence of experimental area effects and the interaction between time and experimental area. This indicated that these factors significantly affected the soil carbon to nitrogen ratio ($p<0.05$). In summary, due to the influence of NA on soil chemical properties such as nitrate nitrogen and ammonium nitrogen under the experimental area effect, the *p*-values were 0.012 and 0.041, respectively. Therefore, the experimental area with NA had a significant impact on most soil chemical properties ($p<0.05$).

Table 3. The correlation between different NA and soil properties (*Indicating $p<0.05$).

Influencing factors	*p*-value						
	Processing effect	Time effect	Experimental area effect	Processing: Time	Processing: Experimental area	Time: Experimental area	Processing: Time: Experimental area
pH value	0.614	0.472	0.595	0.514	0.642	0.561	0.564
NO_3-N(mg/kg)	0.188	0.074	0.012*	0.064	0.112	0.043*	0.049*
NH_4NO_3(mg/kg)	0.075	0.059	0.041*	0.068	0.038*	0.028*	0.044*
Total Nitrogen(g/kg)	0.216	0.044*	0.036*	0.128	0.037*	0.041*	0.111
Soil organic carbon(g/kg)	0.188	0.099	0.048*	0.146	0.157	0.073	0.099
Quick acting phosphorus(g/kg)	0.051	0.070	0.031*	0.062	0.041*	0.051	0.050
Carbon to nitrogen ratio	0.257	0.056	0.043*	0.065	0.156	0.049*	0.055

3.3. Influence of NA on seasonal changes in soil respiration

3.3.1. Seasonal difference analysis of NA on soil respiration rate

Soil respiration rate had no significant seasonal difference under natural nitrogen deposition when the nitrogen deposition concentration was A ($p>0.05$). Soil respiration rate had a significant seasonal difference with the increase of NA concentration when the nitrogen deposition concentrations were B and C ($p<0.05$).

Table 4. Seasonal differences in soil respiration rate under different NA(*Indicating $p<0.05$).

Experimental area	Nitrogen deposition concentration	*p*-value		
		Day 1 to Day 60	Day 60 to Day 120	Day 120 to Day 150
Experimental areas closer to the suburbs	A	0.061	0.068	0.094
	B	0.006*	0.001*	0.026*

	C	0.016*	0.003*	0.031*
Experimental areas far from the suburbs	A	0.051	0.092	0.090
	B	0.040*	0.008*	0.022*
	C	0.031*	0.011*	0.036*

3.3.2. Influence of NA on soil heterotrophic respiration

An in-depth analysis was conducted to analyze the effect of different concentrations of soil NA on soil heterotrophic respiration as shown in Figure 9. In August, both the suburban and exurb experimental areas showed a slight increase in RH caused by nitrogen deposition compared to the nitrogen blank A. However, the RH rates of nitrogen deposition at different concentrations were similar in October and December. In the suburban experimental area of Figure 9 (a), the soil respiration rates of nitrogen deposition concentrations B and C were 1.28±0.12 μmol·m^{-2}·s^{-1} and 1.22±0.01 μmol·m^{-2}·s^{-1} in August. Compared with the soil respiration rate of 1.15±0.13 μmol·m^{-2}·s^{-1} at concentration A at this time, they increased by 11.30% and 6.08%, respectively (p<0.05). In the exurb experimental area in Figure 9 (b), the soil respiration rates of nitrogen deposition concentration B and concentration C in August were 0.94±0.04 μmol·m^{-2}·s^{-1} and 1.04±0.04 μmol·m^{-2}·s^{-1}. Compared with the soil respiration rate of 0.88±0.21 μmol·m^{-2}·s^{-1} at concentration A at this time, it increased by 6.81% and 18.18%, respectively (p<0.05). In summary, different NAs have an impact on the heterotrophic respiration rate of soil [31].

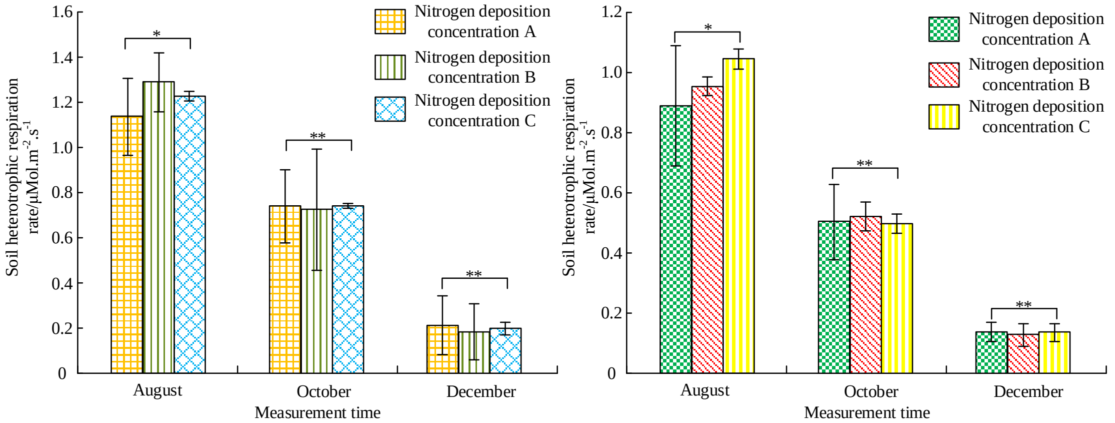

(a) Experimental sites closer to the suburbs (b) Experimental sites far from the suburbs

Figure 9. Influence of NA on soil heterotrophic respiration.(*Indicating p<0.05， **Indicating p>0.05).

3.3.3. Correlation analysis between heterotrophic respiration rate with different NAs and seasons

In the suburban experimental area, when the concentration of nitrogen deposition was A, the Respiration Soil (RS), Respiration Autotrophic (RA), and RH were 0.621, 0.795, and 0.559, respectively, and the seasonal *p*-value was 0.046. This indicated a significant correlation between soil respiration and seasonal changes in the region, especially a high correlation between RH and seasonal changes ($p<0.05$). In the exurb experimental area, under different nitrogen deposition concentration conditions, the seasonal *p*-value was less than 0.05. Therefore, although the values of indices such as RS, RA, and RH were relatively low, the impact of seasonal changes on soil respiration was still obvious ($p<0.05$). Therefore, the seasonal changes of soil respiration among different NA locations and fertilization cycles had obvious differences ($p<0.05$).

Table 5. Correlation analysis between different NA and soil respiration rates with seasons(*Indicating $p<0.05$).

Experimental area	Nitrogen deposition concentration	*p*-value			
		RS	RA	RH	Season
Experimental areas closer to the suburbs	A	0.621	0.795	0.559	0.046*
	B	0.561	0.625	0.492	0.032*
	C	0.349	0.684	0.450	0.021*
Experimental areas far from the suburbs	A	0.297	0.526	0.335	0.049*
	B	0.154	0.449	0.295	0.030*
	C	0.335	0.395	0.264	0.028*

3.3.4. Analysis of factors affecting soil respiration

In Figure 10, the red and black lines represent negative correlation and positive correlation, respectively. The solid and dashed lines indicate significant and insignificant differences, respectively. The addition of nitrogen was positively correlated with total soil nitrogen, with significant differences, and negatively correlated with soil pH, with no significant differences. The total nitrogen in soil was positively correlated with RH with significant difference. RH and RA were positively correlated with RS with significant difference. The pH of soil was significantly negatively correlated with RA. RA was also significantly negatively correlated with RH.

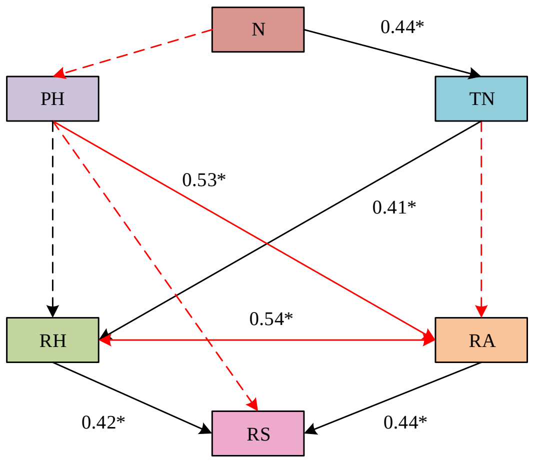

Figure 10. A Structural equation model for analyzing factors influencing soil respiration(**p*<0.05).

Various soil properties were merged into one table to observe the effects of NA on soil respiration and its components in urban forests more intuitively. The correlation table between soil properties and NA is shown in Table 6. NA had a significant impact on soil properties such as temperature, moisture content, total nitrogen, nitrate nitrogen, and ammonium nitrogen, while its impact on other soil properties was not significant [32].

Table 6. Correlation table between soil properties and nitrogen addition.

Experimental area		*p*-value of soil properties							
		Soil properties				Soil respiration		Soil environmental conditions	
		pH value	Total nitrogen	Nitrate nitrogen	Ammonium nitrogen	Autotrophic respiration	Heterotrophic respiration	Moisture content	Temperature
Experimental areas closer to the suburbs	A	0.499	0.049*	0.042*	0.011*	0.795	0.559	0.042*	0.039*
	B	0.481	0.035*	0.035*	0.034*	0.625	0.492	0.026*	0.008*
	C	0.464	0.022*	0.003*	0.035*	0.684	0.45	0.031*	0.029*
Experimental areas far from the suburbs	A	0.534	0.044*	0.016*	0.029*	0.526	0.335	0.004*	0.024*
	B	0.491	0.006*	0.029*	0.034*	0.449	0.295	0.016*	0.036*
	C	0.381	0.019*	0.031*	0.041*	0.395	0.264	0.044*	0.049*

4. DISCUSSION

From the correlation analysis between heterotrophic respiration rates with different NA and seasons, it can be seen that NA has a relatively low response in the short term, indicating that the effect of NA on soil respiration is not significant in the short term. The reason for this finding may be that it takes time for microorganisms and plant roots to adapt to increased nitrogen. The

adjustment of microbial community structure, the change of enzyme activity, and the growth of plant roots all require a certain time period in response to the increase of external nitrogen, so significant changes in respiration rate may not be observed in the short term. The seasonal changes have a significant impact on soil respiration, indicating that seasonal factors such as temperature and humidity play a more significant role in regulating soil respiration. Although the interaction of NA does have an impact on soil respiration, the impact of NA is relatively small compared to the impact of seasonal changes on soil respiration (p<0.05). This may be due to the greater fluctuation range of environmental factors brought about by seasonal changes, and the more significant interference on soil biological activities. In addition, as an important interface of soil-atmosphere carbon exchange, the growth status, species composition and physiological status of above-ground plant communities were both regulated by nitrogen supply and seasonal changes, which indirectly affected the response of soil respiration to NA. The above ground plant community might play an important role in regulating the response of soil respiration to nitrogen deposition[33]. This study found that temperature has a relatively small impact on soil respiration. This is because NA can enhance soil microbial metabolism and plant root activity. The heat released by metabolism and root respiration affects soil temperature. Hu J's research team used meta-analysis to compare 100 microbial data pairs and found that NA reduced microbial carbon utilization by 12%, indicating that NA affects microbial metabolism [13]. This result is consistent with the research conclusion of this article. Li X's research team found that under different NA, the fine root yield in the lower canopy of temperate deciduous forests is twice that of understory NA [14]. These studies indicate that NA has a significant impact on soil respiration, root biomass, microbial metabolism, and other aspects. However, the research results of this article show that low nitrogen has no significant effect on soil temperature, while high nitrogen has a corresponding increase and decrease in soil temperature. NA can improve soil moisture conditions, enhance soil water persistence, promote soil water and heat functions, enhance soil permeability, and promote soil water transport. This may be related to the amount of nitrogen added and the chemical changes of soil. Low nitrogen treatment may not be enough to cause significant changes in soil temperature, while high nitrogen treatment may release a lot of heat through promoting microbial activity and decomposition of organic matter, thus affecting soil temperature. The above research results of this article are also

similar to their conclusions. In a one-year experiment, significant differences were observed in soil organic carbon under the influence of the experimental area effect, indicating that the experimental area has a significant impact on soil organic carbon content. This is because temperature and precipitation can affect the growth and decomposition rate of plants, thus the experimental area has a significant impact on soil organic carbon (p<0.05). Under the experimental area effect, the p-value of available phosphorus is 0.031. The p-value under the interaction effect between processing and experimental area is 0.041. Therefore, under the influence of the experimental area effect and the interaction effect between treatment and experimental area, the difference in available phosphorus is significant (p<0.05). When the nitrogen deposition concentrations are B and C, although the RS, RA, and RH values are lower than the concentration A, the p-values are all less than 0.05. Therefore, seasonal changes still significantly affect soil respiration under nitrogen deposition concentration (p<0.05). The application of nitrogen treatment in soil promoted root respiration and microbial respiration in urban forest soil. With the increase of fertilization and seasonal changes, the trend of the effect of nitrogen treatment on soil was to promote microbial respiration in urban forest soil and inhibit root respiration. Therefore, the response of soil respiration components to NA was non-linear. This may be related to the diversity and complexity of the soil biome. The response of different microbial populations and plant roots to nitrogen is different, and the interaction between them may also affect the overall performance of soil respiration. In addition, the disturbance of soil ecosystems by human activities may also lead to the increased complexity of the NA response of soil respiration components. which could not be ruled out that the influence of human activities on them led to the cancellation of the responses of each component to nitrogen treatment. Therefore, it can be concluded that nitrogen is not a limiting factor for soil. Due to the balance ability of the ecosystem, NA enhances the activity of microorganisms in the soil, thereby enhancing soil respiration.

5. CONCLUSION

A nitrogen deposition simulation experiment was conducted in *Quercus acutissima* forests in the suburbs of Hefei to explore the effects of NA on soil respiration and its components in urban forests. The experimental results showed that low-concentration nitrogen supplementation (50 g

N·m^{-1}) significantly promoted soil respiration in summer, increasing soil respiration rate by about 11% (p<0.05), but decreasing soil respiration rate by about 6% (p<0.05) in winter, with a range of 5% to 20%. The addition of high concentration nitrogen (100 g N·m-1) significantly increased soil temperature by about 12% in summer (p<0.05), but decreased it by about 13% in winter (p<0.05), which revealed the seasonal regulation effect of nitrogen concentration on soil temperature. Nitrogen addition significantly increased soil total nitrogen and nitrate nitrogen contents (p<0.05), but had no significant effect on soil pH value. Structural equation model analysis showed that nitrogen addition directly or indirectly regulated soil heterotrophic respiration and autotrophic respiration by affecting soil nutrient status. Under the condition of natural nitrogen deposition (N concentration A), the seasonal difference of soil respiration rate was not significant (p>0.05), but with the increase of N concentration to B and C, the seasonal difference became significant (p<0.05). In conclusion, compared with untreated control group, nitrogen supplementation (50 g N·m^{-1} and 100 g N·m^{-1}) significantly increased soil respiration rate by about 11% and 7.36% in summer (p<0.05), and decreased by about 6% and 1.96% in winter (p<0.05), respectively. Nitrogen addition is most likely to affect soil respiration by promoting soil microbial metabolism and plant root activities. At the same time, changes in soil properties and decomposition rate of organic matter caused by nitrogen addition also indirectly affect soil temperature, which in turn affects soil respiration. Future research should comprehensively evaluate the impact of nitrogen deposition on soil respiration and ecosystems, in order to better understand and predict the response and adaptation mechanisms of soil ecosystems in the process of urbanization. In addition, in future studies, it is necessary to include weather parameters such as rainfall and temperature into the observation range, and in-depth analysis of the interaction mechanism between these parameters and soil respiration and soil properties. The comprehensive assessment of the combined effects of nitrogen addition and weather factors is expected to reveal the dynamic change law of soil ecosystem in the process of urbanization, and provide a more scientific and comprehensive theoretical basis and practical guidance for urban greening and ecological protection.

References

[1] J. Karst, J. Wasyliw, J. D. Birch, J. Franklin, S. X. Chang, and N. Erbilgin, “Long-term nitrogen addition does not sustain host tree stem radial growth but doubles the abundance of high-biomass ectomycorrhizal fungi,” Global Change Biology, vol. 27, no. 17, pp. 4125-4138, May 2021, https://doi.org/10.1111/gcb.15713.

[2] Y. Yang, H. Cheng, H. Gao, and S. An, “Response and driving factors of soil microbial diversity related to global nitrogen addition,” Land Degradation & Development, vol. 31, no. 2, pp. 190-204, September 2020, https://doi.org/10.1002/ldr.3439.

[3] R. Wang, B. Bicharanloo, M. B. Shirvan, T. R. Cavagnaro, Y. Jiang, C. Keitel, and F. A. Dijkstra, “A novel 13C pulse-labelling method to quantify the contribution of rhizodeposits to soil respiration in a grassland exposed to drought and nitrogen addition,” New Phytologist, vol. 230, no. 2, pp. 857-866, November 2021, https://doi.org/10.1111/nph.17118.[4] C. Wang, R.

[4]Zhang, L. Vilonen, Y. Qu, X. Fu, B. Shi, and W. Sun, “Grazing and nitrogen addition restructure the spatial heterogeneity of soil microbial community structure and enzymatic activities,” Functional Ecology, vol. 35, no. 12, pp. 2763-2777, September 2021, https://doi.org/10.1111/1365-2435.13926.

[5] Ding, W. Cong, W. F. Lambers, H, “Plant phosphorus-acquisition and-use strategies affect soil carbon cycling,” Trends in Ecology & Evolution, vol. 36, no.10, pp.899-906, July 2021, https://doi.org/10.1016/j.tree.2021.06.005.

[6] Hu W, Ran J, Dong L, Du, Q., Ji, M., Yao, S.& Deng, J. "Aridity-driven shift in biodiver sity–soil multifunctionality relationships," Nature Communications, vol. 12, no. 1, pp. 5350-5357, September 2021, https://doi.org/10.1038/s41467-021-25641-0.

[7] Picchio R, Mederski P S, Tavankar F, “How and how much, do harvesting activities affect forest soil, regeneration and stands?," Current forestry reports, vol. 6, no. 2, pp. 115-128, April 2020, https://doi.org/10.1007/s40725-020-00113-8

[8] W. Dou, M. Delgado-Baquerizo, D. Revillini, & G. J. Kidron, “Dryland nitrogen deposition induces microbiome-driven increases in biocrust respiration and losses of soil carbon” Land Degradation & Development, vol. 35, no. 2, pp. 647-658, October 2023, https://doi.org/10.1002/ldr.4942

[9] Huang W, Ye C, Hockaday W C, & Hall, S. J. "Trade-offsinsoilcarbonprotectionmechanisms underaerobicandanaerobicconditions,"GlobalChangeBiology,vol.26,no.6,pp.3726-3737,March2020,https://doi.org/10.1111/gcb.15100.

[10] Koudahe K, Allen S C, Djaman K. "Criticalreviewoftheimpactofcovercropsonsoilproperties," InternationalSoilandWaterConservationResearch,vol.10,no.3,pp.343-354,March2022,https://doi.org/10.1016/j.iswcr.2022.03.003.

[11] L. Zhou, X. Zhou, B. Zhang, M. Lu, Y. Luo, L. Liu, & B. Li,"Different responses of soil respiration and its components to nitrogen addition among biomes: a meta-analysis" Global change biology, vol. 20, no. 7, pp. 2332-2343, 2014

[12] Y. Du, H. Han, Y. Wang, M. Zhong, D. Hui, S. Niu, & S. Wan, "Plant functional groups regulate soil respiration responses to nitrogen addition and mowing over a decade" Functional Ecology, vol. 32, no.4, pp. 1117-1127, 2018

[13] Y. Han, J. Feng, M. Han, and B. Zhu, "Responses of arbuscular mycorrhizal fungi to nitrogen addition: a meta-analysis," Global Change Biology, vol. 26, no. 12, pp. 7229-7241, September 2020, https://doi.org/10.1111/gcb.15369.

[14] J. Hu, C. Huang, S. Zhou, and Y. Kuzyakov, "Nitrogen addition to soil affects microbial carbon use efficiency: Meta-analysis of similarities and differences in 13C and 18O approaches," Global Change Biology, vol. 28, no. 16, pp. 4977-4988, May 2022, https://doi.org/10.1111/gcb.16226.

[15] X. Li, C. Zhang, B. Zhang, D. Wu, Y. Shi, W. Zhang, and S. Fu, "Canopy and understory nitrogen addition have different effects on fine root dynamics in a temperate forest: implications for soil carbon storage," New Phytologist, vol. 231, no. 4, pp. 1377-1386, May 2021, https://doi.org/10.1111/nph.17460.

[16] M. Molaei Arpnahi, M. H. Salehi, M. Karimian Egbal, and Z. Mosleh, "Effect of land-use change on some physical and chemical indices of soil quality in the Bazoft Region, (Chaharmahal-Va-Bakhtiari Province)," Water and Soil, vol. 34, no. 3, pp. 707-720, October 2020, https://doi.org/10.5194/soil-6-53-2020.

[17] M. Molaei Arpnahi, M. H. Salehi, M. Karimian Egbal, and Z. Mosleh, "Effect of land-use change on some physical and chemical indices of soil quality in the Bazoft Region,

(Chaharmahal-Va-Bakhtiari Province)," Water and Soil, vol. 34, no. 3, pp. 707-720, October 2020, https://doi.org/10.22067/JSW.V34I3.84914

[18] Huiguang J, & Yeyun, L. "Theeffectsofdifferenttypesofmulchonsoilpropertiesandteaproduction andquality,"JournaloftheScienceofFoodandAgriculture,vol.100,no.14,pp.5292-5300,June2020,https://doi.org/10.1002/jsfa.10580.

[19] Chen W, Meng H, Song H, & Zheng, H. "Progress in dust modelling, global dust budgets, and soil organic carbon dynamics," Land, vol. 11, no. 2, pp. 176-183, January 2022, https://doi.org/10.3390/land11020176.

[20] B. Sridhar, R. C. Wilhelm, S. J. Debenport, T. J. Fahey, D. H. Buckley, and C. L. Goodale, "Microbial community shifts correspond with suppression of decomposition 25 years after liming of acidic forest soils," Global Change Biology, vol. 28, no. 18, pp. 5399-5415, June 2022, https://doi.org/10.1111/gcb.16321.

[21] E. A. Clippard, S. I. Haruna, P. S. Curtis, C. Clay, B. Bond-Lamberty, K. Mathes, and C. M. Gough, "Decadal forest soil respiration following stem girdling," Trees, vol. 36, no. 6, pp. 1943-1949, August 2022, https://doi.org/10.1007/s00468-022-02340-x.

[22] L. Sherman and M. D. Coleman, "Forest soil respiration and exoenzyme activity in western North America following thinning, residue removal for biofuel production, and compensatory soil amendments," GCB Bioenergy, vol. 12, no. 3, pp. 223-236, January 2020, https://doi.org/10.1111/gcbb.12668.

[23] Landis J D, Obrist D, Zhou J, Renshaw, C. E., McDowell, W. H., Nytch, C. J & Taylor, V. F. "Quantifying soil accumulation of atmospheric mercury using fallout radionuclide chronometry," Nature Communications, vol. 15, no. 1, pp. 5430-5437, June 2024, https://doi.org/10.1038/s41467-024-49789-7.

[24] Gavrilescu M. "Water, soil, and plants interactions in a threatened environment," Water, vol. 13, no. 19, pp. 2746-2751,September 2021, https://doi.org/10.3390/w13192746.

[25] Ngao et al. 2007, Savage et al. 2018. Ngao, J., Longdoz, B., Granier, A., & Epron, D. (2007). Estimation of autotrophic and heterotrophic components of soil respiration by trenching is

sensitive to corrections for root decomposition and changes in soil water content. Plant and soil, vol. 301, pp. 99-110.

[26] Savage, K. E., Davidson, E. A., Abramoff, R. Z., Finzi, A. C., & Giasson, M. A. (2018). Partitioning soil respiration: quantifying the artifacts of the trenching method. Biogeochemistry, vol. 140, pp. 53-63.

[27] A. Guo, X. Zuo, S. Zhang, Y. Hu, P. Yue, P. Lv, and Q. Yu, "Contrasting effects of plant inter-and intraspecific variation on community trait responses to nitrogen addition and drought in typical and meadow steppes," BMC Plant Biology, vol. 22, no. 1, pp. 90-103, March 2022, https://doi.org/10.1186/s12870-022-03486-z.

[28] Han L, Zhang B, Chen L, Feng, Y., Yang, Y., & Sun, K. "Impactofbiocharamendmentonsoilaggregationvariedwithincubationdurationandbiocharpyrolysistemperature,"Biochar,vol.3,no.3,pp. 339-347,May2021,https://doi.org/10.1007/s42773-021-00097-z.

[29] Ding X, Li G, Zhao X, Lin, Q., & Wang, X. "Biocharapplicationsignificantlyincreasessoilorganiccarbonunderconservationtillage:an11-yearfieldexperiment,"Biochar,vol.5,no.1,pp.28-32,May 2023,https://doi.org/10.1007/s42773-023-00226-w.

[30] H. Zhu, J. Zhao, and L. Gong, "The morphological and chemical properties of fine roots respond to nitrogen addition in a temperate Schrenk's spruce (Picea schrenkiana) forest," Scientific Reports, vol. 11, no. 1, pp. 3839-3845, February 2021, https://doi.org/10.1038/s41598-021-83151-x.

[31] M. Volk, M. Suter, A. L. Wahl, and S. Bassin, "Subalpine grassland productivity increased with warmer and drier conditions, but not with higher N deposition, in an altitudinal transplantation experiment," Biogeosciences, vol. 18, no. 6, pp. 2075-2090, March 2021,.https://doi.org/10.5194/bg-18-2075-2021

[32] Augusto L, Boča A. "Tree functional traits, forest biomass, and tree species diversity interact with site properties to drive forest soil carbon," Nature Communications, vol. 13,no.1,pp. 1097-1099, March 2022, https://doi.org/10.1038/s41467-022-28748-0

[33] X. Liang, T. Zhang, X. Lu, D. S. Ellsworth, H. BassiriRad, C. and Q. Ye, "Global response patterns of plant photosynthesis to nitrogen addition: A meta-analysis," Global Change Biology, vol.26, no. 6, pp. 3585-3600, March 2020, https://doi.org/10.1111/gcb.15071
