# Supplementary material for: Influence of nitrogen addition on soil respiration and soil properties in urban forests in Hefei city in China
Source: PLoS One. 2025 Jun 25;20(6):e0324615. doi: 10.1371/journal.pone.0324615 (PMC12192109; doi:10.1371/journal.pone.0324615)
Supplement: Attachment — Submitted filename: reply to editor and reviewer.doc [file pone.0324615.s005.doc]

Journal Requirements:

Please review your reference list to ensure that it is complete and correct. If you have cited papers that have been retracted, please include the rationale for doing so in the manuscript text, or remove these references and replace them with relevant current references. Any changes to the reference list should be mentioned in the rebuttal letter that accompanies your revised manuscript. If you need to cite a retracted article, indicate the article’s retracted status in the References list and also include a citation and full reference for the retraction notice.

Reply: Thank you very much for your comments. After careful review, the reference list was complete and correct, and we did not cite the retracted papers.

Review Comments to the Author

Please use the space provided to explain your answers to the questions above. You may also include additional comments for the author, including concerns about dual publication, research ethics, or publication ethics. (Please upload your review as an attachment if it exceeds 20,000 characters)

**Reviewer #1:**

The authors made efforts and addressed most of my concerns. I find that English/presentation still could be further improved. For example, both past tense and present tense are still used to describe the results in this study and in the Materials and Methods section. A few suggest changes are listed for the abstract. I recommend minor revision.

Reply: Thank you very much for your comments. The laguage of the manuscript has been improved by scholars who have published high-level English academic papers.

L13: change “low-concentration nitrogen” to “low nitrogen addition rate”? replace all concentration to addition?

Reply: According to your kind suggestions, “low-concentration nitrogen” was changed to “low nitrogen addition rate”.

L14: decreased it

Reply: According to your kind suggestions, the sentence was changed to “...but decreased it by about 6% in winter...”.

L15: delete “and the results were significantly different (*p*<0.05)”?

Reply: According to your kind suggestions, the sentence “and the results were significantly different (*p*<0.05)” was deleted.

L17: decrease significantly

Reply: According to your kind suggestions, the sentence was changed to “...and significantly decreased soil temperature by about 13% in winter...”.

L19: change “Under these conditions” to “As a result”? delete the

Reply: According to your kind suggestions, “Under these conditions” was changed to “As a result”.

L20: “high concentration nitrogen” is not a current term.

Reply: According to your kind suggestions, “concentration” was changed to “addition”.

L22-23. Had. Delete “the coefficient of determination. R2 is close to 1”.

Reply: According to your kind suggestions, the sentence was changed to “Soil moisture content and temperature had significant effects on...”, and “the coefficient of determination. R2 is close to 1” was deleted.

L25-28: This sentence needs to be revised. There is also a logic issue here. How could N addition influence soil heterotrophic and autotrophic respirations by affecting soil respiration, as soil respiration is the total of them?

Reply: Thank you very much for your comments. Please see the revisions as follows:

Structural equation model analysis showed that nitrogen addition can promote soil microbial activity, release heat, thereby increasing soil temperature, affecting soil respiration rate, soil moisture, soil total nitrogen, and nitrate nitrogen content, directly or indirectly regulating soil heterotrophic respiration and autotrophic respiration.
L28. Missing “.”

Reply: Thank you very much for your comments. “.” has been added.

L164-165: NH4NO3: subscripts. May be errors?

Reply: NH_4_NO_3_.

Fig. 1: Experimental design. It seems that Latin Square design was used in this study. But it is not clear whether this design was considered for ANOVA.

Reply: Thank you very much for your comments. The Latin square design used in this experiment can be used for analysis of variance. This section has been added to the paper. Please see the explanation as follows:

The experiment adopted a Latin square design, where the levels of each factor were systematically assigned to the experimental units, thus minimizing error variation and improving the accuracy of ANOVA.

Reviewer #2:

There are some small issues that remain to be clarified.

- Lines 19-20: units are wrong; missing per unit of surface (µmol m-2 s-1)

Reply: Thank you very much for your comments. The unit has been corrected.

- Line 23: determination R2

Reply: As suggested by reviewer 1, “determination R2” was deleted.

- Line 58 – 59: Nitrogen addition

Reply: Thank you very much for your comments, it was changed to “Nitrogen addition (N-addition)”.

- Line 72: Hu et al.

Reply: Thank you very much for your comments. It was changed to “Hu et al. compared 100 microbial data pairs using meta-analysis methods to study the carbon utilization efficiency of microorganisms [14].”

- Lines 96 - 99: This sentence is similar to the one in lines 110-113; it would be convenient to unify both in a single paragraph.

Reply: According to your kind suggestions, it was unified in a single paragraph.

- Line 124: kilometers

Reply: According to your kind suggestions, it was changed to “kilometers”.

- Line 135: 18.52 kg ·ha-1·yr-1.

Reply: According to your kind suggestions, it was changed to “18.52 kg·ha^-1^·yr^-1^”.

- Line 165: NH4NO3

Reply: According to your kind suggestions, it was changed to “NH_4_NO_3_”.

- Line 175: Is the soil brought to the same bulk density as the soil was before excavation? How is it restored to its original structure? Some explanation in this regard should be provided.

Reply: Thank you very much for your comments. After removing the root system, optimize the use of excavated soil for backfilling, measure various indicators of the backfilled soil, and restore the soil to its original structure by adding organic matter or drip irrigation. The soil's bulk density remains the same as before. When restoring the original structure, the organic matter content, soil moisture, acidity, and alkalinity measured by the original structure are added with corresponding organic matter and nutrients to restore the soil to its original structure.

- Lines 199 – 202: Revise this sentence because neither porosity, soil particle size, soil moisture nor bulk density are chemical properties of soil; they are physical properties.

Reply: Thank you very much for your comments. The chemical properties have been changed to physical properties.

- Line 212: Check this data because it seems strange that the soil has 80% organic carbon.

Reply: I am so sorry for the mistake, the sentence was changed to “The soil type in this region is sandy loam soil containing 5% particulate organic carbon”.

- Lines 222 – 223: although the two soil were similar texture, they showed significant differences in soil thickness, nutrient content, microbial community structure, and pH value

Reply: That’s right, from Table 1, although the two soil were similar texture, they showed significant differences in soil thickness, nutrient content, microbial community structure, and pH value.

- 288: Results

Reply: Thank you very much for your comments. The word was changed to “results”.

- Figure 2: y-axis legend: mol m-2 s-1 (also in figure 8).

Reply: Thank you very much for your comments. The y-axis legend in Figures 2 and 8 has been revised.

- Line 341: figure b y-axis: Soil temperature C

Reply: Thank you very much for your comments. The y-axis unit in Figure 3b has been revised.

- Line 353: Table 2. Columns 3 (Water content) and 4 (Soil moisture), what is the difference between them?

Reply: Thank you very much for your comments. Soil moisture content refers to the direct reflection of soil moisture status by the ratio of the actual water content in the soil to the dry weight of the soil. Soil moisture refers to the ratio of the moisture content in the soil to the dry weight of the soil, focusing on reflecting the dynamic changes in soil moisture and its interrelationships with other soil properties.

- Line 381: figure 4, y-axis: pH

Reply: Thank you very much for your comments. The y-axis unit in Figure 4 has been revised.

- Table 4: NO3NH4 or NH4-N?

Reply: NH_4_-N.
