# Supplementary material for: Influence of nitrogen addition on soil respiration and soil properties in urban forests in Hefei city in China
Source: PLoS One. 2025 Jun 25;20(6):e0324615. doi: 10.1371/journal.pone.0324615 (PMC12192109; doi:10.1371/journal.pone.0324615)
Supplement: Attachment — Submitted filename: Reply to editors and reviewers.doc [file pone.0324615.s006.doc]

Journal Requirements:

Please review your reference list to ensure that it is complete and correct. If you have cited papers that have been retracted, please include the rationale for doing so in the manuscript text, or remove these references and replace them with relevant current references. Any changes to the reference list should be mentioned in the rebuttal letter that accompanies your revised manuscript. If you need to cite a retracted article, indicate the article’s retracted status in the References list and also include a citation and full reference for the retraction notice.

Reply: Thank you very much for your comments. The reference list is complete and correct, and we do not have cited papers that have been retracted.

Additional Editor Comments:

The revised version has been improved a lot. But the manuscript still has some problems as suggested by the two reviewers. The authors should respond to the comments of the reviewers one by one and revise the manuscript accordingly.

Reply: Thank you very much for your comments. We have carefully revised the paper according to the reviewer’s comments. We believe that after revision, it can meet the requirements of the reviewers.

Review Comments to the Author

Please use the space provided to explain your answers to the questions above. You may also include additional comments for the author, including concerns about dual publication, research ethics, or publication ethics. (Please upload your review as an attachment if it exceeds 20,000 characters)

Reviewer #1:

The manuscript still has many grammatic and presentation issues. Both past tense and present tense are still used to describe the methods in the Materials and Methods section and results in this study. I found that ANOVA might not be correctly conducted, as one-way ANOVA was used (L276), but Latin Square design was used in this study (L171). There are also many other issues such as units and style. I just listed a few below. The authors need to carefully check the whole manuscript and make substantial revision.

Reply: Thank you very much for your comments. The whole manuscript has been carefully checked and the grammatic and presentation issues have been adrressed by scholars who have published high-level English papers. The description of the Latin square design has been revised. As follows:

The Latin square design was used in the experiment. By constructing a square matrix, the variation between the horizontal and in-line unit groups was separated from the experimental error. Therefore, the experimental error was smaller than the random unit group design, and the experimental accuracy was higher.

Specific comments:

L20: high nitrogen addition

Reply: Thank you very much for your comments. The sentence has been changed to “The results indicated that the high nitrogen addition had a significant effect on soil respiration rate”.

L22-23: “, the coefficient of determination”: Delete this part.

Reply: According to your kind suggestion, this part has been deleted.

L25: change “can promote” to promoted

Reply: According to your kind suggestion, "can promote" has been changed to "promoted".

L25-28: This sentence is too long. Please revise it.

Reply: Thank you very much for your comments. The sentence has been revised to “Structural equation modeling analysis shows that nitrogen addition promoted soil microbial activity, releases heat, and thus increases soil temperature. At the same time, this method affects soil respiration rate, soil moisture, soil total nitrogen, and nitrate nitrogen content, directly or indirectly regulating soil heterotrophic respiration and autotrophic respiration.”

Please check units and use them consistently. For example, meter or m, Kg /hm2 or kg hm-2.

Reply: Thank you very much for your comments. The units in this paper have been unified as “m” and “kg/hm^2^”

L113: km or kilometers?

Reply: Thank you very much for your comments. The sentence has been revised to “According to the distance (50 km) from the city center...”

L116: mm?

Reply: Thank you very much for your comments. The sentence has been revised to “The experimental area belongs to a subtropical monsoon climate, with an average annual precipitation of about 1000 mm.”

L137: why 10 m not 10 meters?

Reply: Thank you very much for your comments. 55 m × 55 m is the area of the experimental area. If it has been clearly defined as the length unit, it is redundant to reuse the complete unit name "m".

L145: “Licensing Agency: Nanjing Forestry University)”: I don’t understand this part. Delete it.

Reply: According to your kind suggestion, this part has been deleted.

L150-151: why both g per square meter per year and g m-2 yr-1 were used?

Reply: Thank you very much for your comments. This unit has been unified as “g·m^-2^·yr^-1^”.

Please use past tense to describe the methods and results of this study.

Reply: Thank you very much for your comments. The past tense to describe the methods and results of this study.

L162: After removing …

Reply: Thank you for your comments. The sentence has been revised to “When removing soil roots by digging trenches, the pH value, organic matter content, soil temperature, and humidity of the soil were first measured. After removing the root system, the study optimizes the use of excavated soil for backfilling, measures various indicators of the backfilled soil, and restores the soil to its original structure by adding organic matter or drip irrigation.”

L163: change are to were.

Reply: Thank you very much for your comments. 'are' has been changed to 'were'.

L163-171: please change to past tense.

Reply: Thank you very much for your comments. It has been changed to past tense.

L172: change are to were.

Reply: Thank you very much for your comments. 'are' has been changed to 'were'.

L172: “where the levels of each factor are systematically assigned to the experimental units,”: I don’t think this is a correct description for Latin square design.

Reply: Thank you very much for your comments. The description of the Latin square design has been modified. As follows:

The Latin square design was used in the experiment. By constructing a square matrix, the variation between the horizontal and in-line unit groups was separated from the experimental error. Therefore, the experimental error was smaller than the random unit group design, and the experimental accuracy was higher.

L200: “Soil temperature was … using the Li-8100 respirator”: which sensor was used for soil temperature measurement? Li-8100 is for soil CO2 measurements, not temperature.

Reply: Thank you very much for your comments. The soil temperature measuring instrument is 109 soil temperature sensor.

L228: “A respiration apparatus (Li-8100 produced by Li Cor in the United States)”: This is not a correct description for soil respiration instrument. Please check a published papers on the use of Li-8100 and company.

Reply: Thank you very much for your comments. This sentence has been revised to “Measure RS on soil ring using soil respiration analyzer (L-8100A, USA).”

L273-273: this sentence was duplicated (L270-271).

Reply: Thank you very much for your comments. The duplicated sentence has been deleted.

L269-283: The data analysis is a mess and needs to be totally re-organized.

Reply: Thank you very much for your comments. This part has been rewritten. As follows:

During data analysis, Excel software is used to input the collected data, conduct preliminary sorting and basic descriptive statistical analysis. Then the data were tested for normality and homogeneity of variance, and the F value, *p* value, mean deviation and confidence interval of the data were analyzed. SPSS software was used to analyze the correlation, regression and variance of the data. And to verify the significant effects of different N-addition treatments on RS rate and other parameters, one-way ANOVA was performed, and Least Significant Difference (LSD) was used for multiple comparisons to determine whether the differences among treatments were statistically significant (*p*<0.05). Finally, MATLAB software is used to model and visualize the data.

L284: 3. Results. Delete analysis.

I did not check the other parts. The authors need to thoroughly check the whole manuscript.

Reply: Thank you very much for your comments. The analysis in conclusion section has been deleted. And other parts of the manuscript have been checked and modified.

Reviewer #2:

I thank the authors for their responses to my comments and doubts about some aspects of their work. After this first review, a few minor changes are listed:

- Lines 19-20: The units are not correct. Please, use the following: µmol m-2 s-1.

Reply: Thank you very much for your comments. The units have been modified to “µmol m^-2^ s^-1^”.

- Line 20: The results indicated that high addition of nitrogen had a…

Reply: Thank you very much for your comments. “high addition of nitrogen” has been changed to “high nitrogen addition”.

- Lines 21-23: Soil moisture content and temperature had significant effects on soil respiration rate, especially in the suburban test site.

Reply: Thank you very much for your comments. This part has been modified. Deleted redundant "the coefficient of determination"

- Lines 105-106: the following hypotheses were proposed: 1) N-addition has different effects 106 on RS in different seasons, and 2) N-addition can affect soil pH a…

Reply: Thank you very much for your comments. This sentence has been changed to “The objective of this study is to investigate the effect of N-addition on forest RS. In order to explore it, the following hypotheses were proposed: 1) N-addition has different effects on RS in different seasons, and 2) N-addition can affect soil pH and soil moisture, thereby affecting RS rate.”

- Line 198: the pH value, soil moisture and nitrogen content of the pre-treated soil sample should be evaluated.

Reply: Thank you very much for your comments. This sentence has been changed to “When the soil temperature drops to 18-25 degrees Celsius, the pH value, soil moisture and nitrogen content of the pre-treated soil sample should be evaluated.”

- Line 268: In this section you should include the factors to be analyzed and the levels of each factor.

Reply: Thank you very much for your comments. This section has been revised to:

During data analysis, Excel software is used to input the collected data, conduct preliminary sorting and basic descriptive statistical analysis. Then the data were tested for normality and homogeneity of variance, and the F value, *p* value, mean deviation and confidence interval of the data were analyzed. SPSS software was used to analyze the correlation, regression and variance of the data. And to verify the significant effects of different N-addition treatments on RS rate and other parameters, one-way ANOVA was performed, and Least Significant Difference (LSD) was used for multiple comparisons to determine whether the differences among treatments were statistically significant (*p*<0.05). Finally, MATLAB software is used to model and visualize the data.

- Figure 3 a) y-axis: express it as soil moisture or soil water content.

Reply: Thank you very much for your comments. The Y-axis of Figure 3 (a) has been modified to “soil water content”. As follows:

**Figure 3** Influence of N-addition on soil moisture content and temperature in urban forests.

- Table 2: Add units to water content and soil moisture.

Reply: Thank you for your comments. The units of water content and soil moisture have been added in Table 2. Please see details as follows:

**Table 2.** Correlation between the RS rate and soil moisture.

| Experimental area | Nitrogen deposition concentration | Water content(%) | Soil moisture(%) | Parameters | |  |
| --- | --- | --- | --- | --- | --- | --- |
|  |  |  |  | R^2^ | *p-*value | |
| Experimental areas closer to the suburbs | A | 6.8 | 28.1 | 0.993 | 0.042 | |
|  | B | 4.1 | 31.9 | 0.959 | 0.026 | |
|  | C | 7.4 | 26.6 | 0.956 | 0.031 | |
| Experimental areas far from the suburbs | A | 6.9 | 14.0 | 0.632 | 0.004 | |
|  | B | 8.5 | 12.7 | 0.559 | 0.016 | |
|  | C | 5.2 | 15.7 | 0.700 | 0.044 | |

- Lines 351-354: In my opinion, this paragraph is not well understood. Please rewrite it to make clear the difference you make between water content and soil moisture.

Reply: Thank you very much for your comments. It has been rewritten to:

Water content referred to the actual water content in the soil, which was usually expressed in the form of mass or volume. It focused on quantifying the absolute amount of water in the soil and reflected the actual storage of soil water. Soil moisture described the relative index of soil moisture, reflected the relative state of soil moisture, and emphasized the availability of water to plant roots or environmental processes.
