# Supplementary material for: Influence of nitrogen addition on soil respiration and soil properties in urban forests in Hefei city in China
Source: PLoS One. 2025 Jun 25;20(6):e0324615. doi: 10.1371/journal.pone.0324615 (PMC12192109; doi:10.1371/journal.pone.0324615)
Supplement: Attachment — Submitted filename: Reply to Reviewer Comments.doc [file pone.0324615.s004.doc]

Review Comments to the Author

Please use the space provided to explain your answers to the questions above. You may also include additional comments for the author, including concerns about dual publication, research ethics, or publication ethics. (Please upload your review as an attachment if it exceeds 20,000 characters)

Reviewer #1: This study was designed to study the impact of N deposition on urban forest soil respiration and soil properties. The authors selected two sites in a city in China, and measured soil respiration and its components, along with other soil properties under three N addition rates (to simulate N deposition) over one year. They reported these values and found that N additions significantly influence soil respiration but had different impacts in summer and winter. N addition influenced soil nutrient status, thus, influenced soil heterotrophic and autotrophic respiration.

I think this study provides some useful data for soil respiration in urban forests, as these data were relatively less reported compared to natural or managed forests. However, the manuscript was poorly written, with many awkward sentences. It was verbose and lengthy and needed to be thoroughly revised. I strongly suggest the authors to find a native English speaker or an editing company to edit the manuscript. The tables and figures need to be self-explanatory. Detailed captions should be provided. I recommend the authors read some published papers and see how the Results section was written. I recommend Major revision.

Reply: Thank you very much for your comments. The laguage of this paper has been improved by scholars who have published high-level English papers. The tables and figures and their explanations have been revised. The analysis and discussion of the experimental results have been modified. Please see the revisions in the revised paper.

Specific comments:

The manuscript lacks line number and page number. It would help reviewer to review it by adding page and line numbers.

Reply: Thank you very much for your comments. Line number and page number have been added.

Please use past tense to describe the results from this study.

Reply: Thank you very much for your comments. The tense in the description of the results has been changed to past tense.

Abstract:

The major results were not clear. Adding N increased soil nutrients is not new and important. Please provide new and important results from this study.

Reply: Thank you very much for your comments. Some key findings have been provided to the abstract section as follows:

Structural equation model analysis showed that the amount of nitrogen added directly or indirectly regulates soil heterotrophic respiration and autotrophic respiration by affecting soil respiration rate, soil moisture, and soil total nitrogen and nitrate nitrogen content

Change nitrogen concentrations to nitrogen addition rates

Reply: Thank you very much for your comments. The concentrations of nitrogen has been changed to the nitrogen addition rates.

The addition of high concentration nitrogen …: how about the soil respiration change under this condition?

Reply: Thank you very much for your comments. Changes in soil respiration under high nitrogen concentration have been added to the abstract according to your suggestion, as follows:

High nitrogen concentration (15 g·m^-2^·yr^-1^) significantly increased soil temperature by about 12% in summer (*p*<0.05), and significantly decreased soil temperature by about 13% in winter (*p*<0.05), indicating the seasonal regulation effect of nitrogen concentration on soil temperature. Under these conditions, the soil respiration rate was 3.26±0.45 μmol^-2^·s^-1^ in summer and 0.51±0.07 μmol^-2^·s^-1^ in winter. The results indicated that the addition of high concentration nitrogen had a significant effect on soil respiration rate, which increased in summer and decreased in winter.

“… and the results were significantly different”. It is not clear here. Significantly different among N additions?

Reply: Thank you very much for your comments. Here, the effects of low nitrogen concentration (5 g·m^-2^·yr^-1^) and high nitrogen concentration (15 g·m^-2^·yr^-1^) on soil respiration rate were compared, so there was a significant difference (triple difference) between the N additions of the two methods.

The correlation coefficient R2: R2 is not correlation coefficient, but coefficient of determination.

Reply: Thank you very much for your comments. The “correlation coefficient” has been changed to “coefficient of determination”.

Introduction:

At present, some studies believe that NA can promote soil respiration and thus reduce soil carbon

Content: I think the general consensus was that N addition could reduce soil respiration, mostly due to reduction in soil pH.

Reply: I'm sorry for the confusion. The sentence has been changed to “At present, some studies believe that N addition can reduce the pH value of soil, thereby reducing soil respiration”

Han Y analyzed: Was this correctly cited? Han (****)?

“Li Xet” should be “Li X et”

Reply: Thank you very much for your comments. They were changed to:

Han et al.

Li et al.

Host roots?

Reply: Thank you very much for your comments. This sentence was changed to “Li et al. conducted a field experiment using temperate deciduous forests to investigate the effects of different N addition on the dynamics of taproot in temperate forests [15].”

Molaei A M. et al. … to research parameters: land use and land use efficient. Why discuss land use change here?

Reply: Thank you very much for your comments. This content was changed to “Molaei et al. conducted comparative experiments based on four different soil management methods to investigate the effects of soil microbial activity and root respiration on soil respiration in natural forests. The results showed that land use changes from natural forests to other uses had a significant impact on most research parameters. Different land use patterns could affect the percentage of particle size distribution. Natural forests and walnut orchards had higher organic matter content, and due to the higher clay ratio, they had stronger water retention capacity. These factors could promote soil microbial activity and root respiration, thereby increasing soil respiration rate [17]”.

End of introduction: Please add objectives of this study, and hypotheses.

Reply: Thank you very much for your comments. The objectives and hypotheses have been added at the end of the introduction. The addition is as follows:

The objective of this study is to investigate the effect of N addition on forest soil respiration. In order to explore it, the following hypotheses were proposed: Hypothesis 1: N addition has different effects on soil respiration in different seasons. Hypothesis 2: N addition can affect soil pH and soil moisture, thereby affecting soil respiration rate.

The average temperature is maintained: how to maintain temperature?

Reply: Thank you very much for your comments. The average annual temperature in the region is maintained at around 17 ℃, providing an ideal environment for the growth of *Quercus acutissima* Carruth.

NA (Nitrogen addition): NA is not a common abbreviation. I suggest just using “N addition”.

Reply: Thank you very much for your comments. NA has been changed to N addition.

Materials and methods:

Research site:

Two sites were selected. Please use a table to illustrate the similarity and differences between them.

Reply: Thank you very much for your comments. The similarity and differences between the two cites have been added as follows:

**Table 1 Comparision of Soil Properties between Suburban and Outer Suburban Areas**

| Location | pH | Thickness of soil | Nutrient content | Microbiologic population；microflora | Agrotype |
| --- | --- | --- | --- | --- | --- |
| Suburban | 6.5~7.5 | Large thickness | Low nutrient content | Rich in biological content | Sandy soil, clay soil, loam soil |
| Outer Suburban | 3.5~5.5 | Small thickness | Organic matter is high | Water-absorbing bacteria, rhizobia | Sandy soil, clay soil, loam soil |

Fig. 1: This map is not very useful. Please move to Supplemental document.

Reply: Thank you for your comments. Figure 1 has been deleted.

Experimental area design: (how to design an area), change design to selection?

Reply: Thank you for your comments. The “design” has been changed to “selection”.

How were 50 gN m-2yr-1 and 100 g N m-2 yr-1 determined?

Reply: Thank you for your comments. The description of the data here is wrong, it should be 5 g·m^-2^·yr^-1^ and 15 g·m^-2^·yr^-1^, and this data is detailed in the method description. A control group of 0, B group of low nitrogen 5 g·m^-2^·yr^-1^, C group for high nitrogen 15 g·m^-2^·yr^-1^.

“the study area was divided into outer suburbs and outer suburbs.”: ?

Reply: Yes, according to the distance from the urban center, the study area was divided into Suburban and Outer Suburban

“The measured respiration heterotrophic (RH) of the soil is subtracted from the soil environmental measurement value to accurately determine the root respiration value of the soil”: this sentence is not correct. What’s soil environmental measurement value?

Reply: Thank you for your comments. The determination of the root respiration value of the soil has been revised as follows:

Subtracting the measured soil RH from the RS value measured by the respiration meter can accurately determine the root respiration value of the soil.

Dividing the tree roots with plastic cloth: separating the roots?

Reply: Yes, we mean that the root area can be separated by a plastic cloth.

The soil moisture was measured by drying method: Drying method is not a correct term for the method. It is called the gravimetric method.

Reply: Thank you for your comments. “Drying method” has been changed to “gravimetric method”.

“Statistical analysis was conducted by using software such as Excel, SPSS, and MATLAB. First, the collected statistical testing data were organized, using Excel software for data entry, preliminary organization, and basic descriptive statistical analysis. Then, SPSS software was used to perform correlation analysis, regression analysis, and analysis of variance on the data. Finally, the MATLAB software was used to model and visualize the data.” This part is data analysis and should be moved to Data analysis below.

Reply: Thank you for your comments. The data analysis section in the text has been adjusted to Section 2.5.

“LSD (minimum significant difference method)”: Least Significant Difference. Please used the correct term.

Reply: Thank you for your comments."minimum significant difference method" has been modified to "Least Significant Difference". The modifications are shown as follows:

LSD (Least Significant Difference) was used for multiple comparisons to determine whether the differences among treatments were statistically significant (p<0.05).

3. Results

Tables 1 and 2. If p value is shown, there is no need to label * or **. What are factor 1 and factor 2. Please add notes to the tables.

Reply: Thanks for your comments. * in Table 1 and Table 2 has been deleted, and factor 1 and factor 2 are Water content and Soil moisture respectively, which have been modified in Table 1.

**Table 2.** Correlation between the RS rate and soil moisture

| Experimental area | Nitrogen deposition concentration | Water content | Soil moisture | Parameters | | |
| --- | --- | --- | --- | --- | --- | --- |
|  |  |  |  | *R*^2^ | Q_10_ | *p-*value |
| Experimental areas closer to the suburbs | A | 0.068 | 0.281 | 0.993 | 2.05 | 0.042 |
|  | B | 0.041 | 0.319 | 0.959 | 2.11 | 0.026 |
|  | C | 0.074 | 0.266 | 0.956 | 2.00 | 0.031 |
| Experimental areas far from the suburbs | A | 0.069 | 0.140 | 0.632 | 0.97 | 0.004 |
|  | B | 0.085 | 0.127 | 0.559 | 0.86 | 0.016 |
|  | C | 0.052 | 0.157 | 0.700 | 0.18 | 0.044 |

Fig. 10: please add notes for the abbreviations.

Reply: Thank you for your comments. Notes of the abbreviations have been added in this Figure. The modifications are made as follows:

Table 6: I don’t understand these values. How were these correction coefficients calculated between soil properties and N addition (3 values)?

Reply: Thank you for your comments. The values in Table 6 represent the correlation between the amount of nitrogen addition and various soil indicators in different regions. Calculated directly by the SPSS software.

Reviewer #2: This work assesses the effect of N addition (simulating atmospheric N deposition) on soil respiration. The effect of soil properties is also studied. All this in an urban forest. This has been widely studied in non-urban forests and, therefore, the novelty of the work is precisely the location of the study area. In this sense, the work could provide more knowledge of the functioning of the forest ecosystem in these areas subjected to greater anthropic pressure.

I had to make my revision in the file, as the manuscript lacks continuous line numbering and it is impossible to refer to any part of the document without a line number. All these comments and some more are included in the file attached to the authors.

Reply: Thank you very much for your comments. Line numbers have been added to the paper.

General comments:

There are many comments and doubts that should be resolved before the manuscript is published. The material and methods section should be improved, regarding the number of samples used and measurements made, a better description of the soil, some correlation analysis is not well explained; in the results section, there is no table with the measured soil values (only the p-value is used), the properties of the soils of the sampling zones are not contrasted; the discussion of the results requires a greater contrast with the works of other authors.

All these comments and some more are included in the file attached to the authors.

Reply: Thank you for your comments. The paper has been revised according to the attachment content.

- There are no working hypotheses

Reply: Thank you for your comments. The working hypotheses has been listed as follows:

the following hypotheses were proposed: Hypothesis 1: N addition has different effects on soil respiration in different seasons. Hypothesis 2: N addition can affect soil pH and soil moisture, thereby affecting soil respiration rate.

- The doses applied to simulate N deposition are very high compared to those used in other similar work (e.g., see Mo, J., Zhang, W. E. I., Zhu, W., Gundersen, P. E. R., Fang, Y., Li, D., & Wang, H. U. I. (2008). Nitrogen addition reduces soil respiration in a mature tropical forest in southern China. Global Change Biology, 14(2), 403-412).

Reply: Thank you for your comments. The dose of nitrogen deposition has been checked:

Zone B is applied with low nitrogen, and the amount of nitrogen applied per square meter is 5 g/year, that is, 5 g ·m^-2^·yr^-1^. High nitrogen is applied in Zone C at 15 g per square meter per year, that is, 15 g ·m^-2^·yr^-1^.

- I suggest a more thorough review of the bibliography, since there are references closely related to the study that have not been used, and could be useful for a better discussion of the results obtained in this work.

Reply: Thank you for your comments. References have been examined. And added to the relevant references. The addition is as follows:

[23]T. Magura, S. Mizser, R. Horváth, M. Tóth, & G. L. Lövei. “Urbanization impoverishes taxonomic but not functional diversity of the gut microbiota in a forest specialist ground beetle, Carabus convexus,” Scientific Reports, vol.14, no. 1, pp. 25546-25554, October2024, <https://doi.org/10.1038/s41598-024-75864-6>

[34]Y. Yang, T. Li ,P. Pokharel, L. Liu, J. Qiao,Y Wang, & S. X. Chang. “Global effects on soil respiration and its temperature sensitivity depend on nitrogen addition rate,” Soil Biology and Biochemistry, vol.174, no. 3, pp. 108814-108820, October2022, https://doi.org/10.1016/j.soilbio.2022.108814

[35]Z. L. Li, Q. Peng , Y. S. Dong , Y. He, Z. Yan, & Y. Guo. “Response of soil respiration to water and nitrogen addition and its influencing factors: A four-year field experiment in a temperate steppe,” Plant and Soil, vol.471, no. 5, pp. 1-16, = December 2021, https://doi.org/10.1007/s11104-021-05221-3

Specific remarks:

- see attached file

Reply: Thank you for your comments. The paper has been revised according to the attachment content. See below:

Check the units; they do not match those mentioned in the material and methods section.

Reply: Thank you for your comments. The unit has been checked. The modifications are shown as follows:

The experimental results showed that low-concentration nitrogen (5 g·m^-2^·yr^-1^ )

The addition of high concentration nitrogen (15 g·m^-2^·yr^-1^)

“The moisture content in soil mainly affects the diffusion of oxygen and the activity of microorganisms.” The rate of soil respiration increases with moisture content up to a certain value near soil saturation, after which respiration decreases.

Reply: Thank you for your comments. The description has been modified to:

The rate of soil respiration increases with moisture content up to a certain value near soil saturation, after which respiration decreases.

Han et al. Li et al. Molaei et al.

Reply: Thank you for your comments. References in the paper are quoted in the above format.

It is difficult to understand how this paragraph fits into the Introductory section's story line.

Reply: Thank you very much for your comments. It has been changed to:

Molaei et al. conducted comparative experiments based on four different soil management methods to investigate the effects of soil microbial activity and root respiration on soil respiration in natural forests. The results showed that land use changes from natural forests to other uses had a significant impact on most research parameters. Different land use patterns could affect the percentage of particle size distribution. Natural forests and walnut orchards had higher organic matter content, and due to the higher clay ratio, they had stronger water retention capacity. These factors could promote soil microbial activity and root respiration, thereby increasing soil respiration rate [17].

Of what order of magnitude is this N deposition?

Reply: Thank you for your comments. Soil total nitrogen content greater than 0.2%.

It would be convenient to provide the reader with some range of nitrogen deposition values in urban forest....

Reply: Thank you for your comments. Urban forest nitrogen deposition has been added in the text. The addition is as follows:

In addition, nitrogen deposition in urban forests is usually high, in urban forest ecosystems, nitrogen deposition is generally 25-60 kg/hm^2^

What is the distance?

Reply: 50 km.

kilograms per hectare and year?

Reply: Thank you for your comments. The description in the text has been modified. The modifications are made as follows:

The exurb experimental area is located in an area at an altitude of 142 meters, with a local N addition level detection value of 16.43 kg ·ha^-1^·yr^-1^, this means that the amount of nitrogen applied per hectare per year is 16.43 kg.

The suburban experimental area is located at an altitude of 92 meters, with an N addition level of 18.52 kg ·ha^-1^·yr^-1^.

It would be convenient to indicate on the map the selected exurban and suburban areas.

Reply: Thank you for your comments. The map has been deleted.

Is this applied amount of the same order as the soil input of N by atmospheric deposition? It must not be, because the amount applied is 500 kg/ha/year, which is a very high amount; and in the high dose 1000 kg/ha/year...they do not seem usual amounts. could you justify why you use those N doses?

Reply: Thank you for your comments. The dose of nitrogen deposition has been modified. The modifications are made as follows:

Zone B is applied with low nitrogen, and the amount of nitrogen applied per square meter is 5 g/year, that is, 5 g·m^-2^·yr^-1^. High nitrogen is applied in Zone C at 15 g per square meter per year, that is, 15 g·m^-2^·yr^-1^.

Why is this type of fertilizer selected? The chemical forms in which N can reach the soil by atmospheric deposition are probably not those, nor in that proportion, which could lead to different results than those obtained.

Reply: Thank you for your comments. The reason for selecting the NH4NO3 has been added in the text. The addition is as follows:

Ammonium nitrate (NH_4_NO_3_) is a commonly-used inorganic nitrogen fertilizer that is chemically stable, easy to control and standardize, and can ensure the stability and reliability of experimental conditions when simulating nitrogen deposition. Although there may be errors in the nitrogen deposition of NH_4_NO_3_, compared with other nitrogen fertilizers, only NH4NO3 can better simulate the nitrogen deposition in natural environments, making the experimental results more practical. So the simulated nitrogen deposition fertilizer is NH_4_NO_3_.‌

This procedure leads to the disturbance of the soil structure in that area, which may make the respiration measurements in the undisturbed and disturbed areas not so comparable.

Reply: Thank you for your comments. Methods for improving soil structure disturbance have been added in the paper. The addition is as follows:

When removing soil roots by digging trenches, first measure the pH value, organic matter content, soil temperature, and humidity of the soil. After removing the root system, optimize the use of excavated soil for backfilling, measure various indicators of the backfilled soil, and restore the soil to its original structure by adding organic matter or drip irrigation.

When are soil samples taken?

Reply: Thank you for your comments. To ensure the measurement accuracy of rootless soil, 10 days after digging trenches and removing roots, soil samples are collected using a soil drill.

I do not understand this pretreatment of soil samples. Surely an important part of the nitrate is lost in the washing. Half of the N supplied is in the form of nitrate, which is very soluble and highly mobile, so the amount lost during filtration can be considerable

Reply: Thank you for your comments. The soil pretreatment methods have been explained as follows:

When sample preprocessing, first, the soil is screened using a 70 mesh sieve to remove large particle impurities, then light impurities in the soil are blown away using wind power, and finally, magnetic impurities in the soil are removed using magnetic blocks. When removing other salts from the soil, electrodialysis is used to separate specific ions from the soil solution to prevent nitrate removal. These samples are placed in the experimental oven for thorough drying after air drying. Finally, these samples are screened for soil particles through a 70 mesh sieve. The drying temperature of the soil is between 105℃ and 110℃. The drying time of the soil is 24 hours.

what is measured in the filtering?

Reply: Thank you for your comments. The measurements from the filtering process have been added to the paper as follows:

Meanwhile, chemical analysis of the filtrate is carried out by measuring the soil pH value, porosity, soil particle size, soil moisture and bulk density, as well as the pH value of the filtrate during the filtration process to ensure the accuracy and reliability of the data.

On what samples? If you are referring to samples collected and pretreated, what is the temperature measured for?

Reply: Thank you for your comments. When the soil temperature drops to 18-25 degrees Celsius, the pH value, soil moisture content, and nitrogen chemical properties of the pre-treated soil sample should be evaluated.

It would be convenient to provide the soil type according to a reference classification such as the Soil taxonomy or the World Reference Base for Soil Resources.

Reply: Thanks for your comments. The soil type in this region is sandy loam soil containing 80% particulate organic carbon, which has good water retention and permeability. The understory vegetation in the area consists of herbaceous plants and shrubs, with the same level of vegetation coverage in the two selected areas.

What is the percentage of organic matter in the soil in the selected areas?

Reply: Thank you for your comments. The percentage of organic matter in the soil in the selected areas is 80%.

What type of vegetation is present in the understory? Is the degree of cover of this vegetation the same in the two selected areas?

Reply: Thank you for your comments. The understory vegetation in the area consists of herbaceous plants and shrubs, with the same level of vegetation coverage in the two selected areas.

“the study area was divided into outer suburbs and outer suburbs. Although the soil types of the two sites were similar, they showed significant differences in soil thickness, nutrient content, microbial community structure and pH value.”check this sentence

Reply: Thank you for your comments. The sentence has been changed to “According to the distance from the urban center, the study area is divided into suburban and outer suburbs, and the similarities and differences of soil characteristics between suburban and suburban are shown in Table 1. From Table 1, although the soil types of the two sites were similar, they showed significant differences in soil thickness, nutrient content, microbial community structure, and pH value.”

“The exurbs remained relatively natural, and the microbial communities were dominated by saprophytic bacteria and rhizosphere microorganisms.”Has it been determined, or is it information from a reference?

Reply: Thank you for your comments. It is information from a reference as follows:

T.Magura, S. Mizser, R. Horváth, M. Tóth, & G. L. Lövei. “Urbanization impoverishes taxonomic but not functional diversity of the gut microbiota in a forest specialist ground beetle, Carabus convexus,” Scientific Reports, vol.14, no. 1, pp. 25546-25554, October2024, <https://doi.org/10.1038/s41598-024-75864-6>

“humidity”soil moisture

Reply: Thank you for your comments. Replaced 'humidity' with 'soil moisture'

Nothing is said about the measurement of soil temperature, how is it measured?

Reply: Thank you for your comments. The test method of soil temperature has been added to the paper, and the specific content is as follows:

Soil temperature was measured by setting up a soil ring in the experimental area and using the Li-8100 respirator. To ensure the accuracy and consistency of the data, soil temperature was measured every month from August 2022 to July 2023 to collect soil temperature data throughout the year. In addition, soil moisture was also measured simultaneously with soil respiration rate to gain a more complete understanding of the influence of soil environment on soil respiration rate.

On a monthly basis? Why was it not measured on soil respiration measurement days?

Reply: Thank you for your comments. The soil respiration rate varies with the seasons, and monthly measurements can reflect the impact of low measurement frequency on soil respiration rate. In order to ensure the accuracy and consistency of the data, soil moisture and soil respiration were measured at the same time period. In addition, soil temperature is measured monthly from August 2022 to July 2023 to collect soil temperature data throughout the year.

This paragraph is repeated (see end of previous section).

Reply: Thank you for your comments. The repeated paragraph “The content of soil moisture is determined by the drying method. Firstly, the fresh soil brought back to the laboratory is poured into a clean aluminum box. The total weight of the fresh soil and aluminum box is weighed. The soil is then placed in an oven set at 105℃ and dried to a constant weight. The total weight of the dry soil and aluminum box is weighed. Finally, the aluminum box is separately weighed to determine the soil moisture content.” has been removed.

What extract do you use, at what concentration?

Reply: Thank you for your comments. The content of nitrate nitrogen and ammonium nitrogen in soil leached was measured with 0.5mol/L K_2_SO_4_ extractant using a flow injection apparatus.

“The analysis of total soil nitrogen is determined using a 3000 element analyzer.”remove“3000”.

Reply: Thank you for your comments."3000" was removed.

What extract do you use, at what concentration?

Reply: Thank you for your comments. Extracts have been added in the paper. The addition is as follows:

The analysis of total soil nitrogen was determined using a element analyzer. A conductivity meter was used to measure the conductivity of soil solution.

Delete this paragraph; it is in the next section.

Reply: Thank you for your comments. This paragraph has been deleted.

The comparison between zones is missing; nothing is said about the exurban zone (Figure 3b).

Reply: Thank you for your comments. The description of this Figure has been added to the paper. The addition is as follows:

In the suburban experimental area, the changes in RS rate at nitrogen deposition concentrations A, B, and C were roughly the same as those in the suburbs. Compared with the RS rate at nitrogen deposition concentration A, the RS rates at nitrogen deposition concentrations B and C both increased in August. In October, there was a decline.

remove bold type

Reply: Thank you for your comments. Bold type has been removed.

“.In January, the moisture content of nitrogen deposition concentrations”check the sentence

Reply: Thank you for your comments. Sentences have been examined and modified. The modifications are made as follows:

In January, the soil moisture content with nitrogen deposition concentrations of A, B, and C

volumetric? gravimetric?

Reply: Gravimetric.

“In August, the moisture content of nitrogen deposition concentrations A, B, and C were 13.22%, 7.91%, and 13.63%, respectively”check the sentence

Reply: Thank you for your comments. Sentences have been examined and modified. The modifications are made as follows:

In August, the soil moisture content with nitrogen deposition concentrations of A, B, and C were 13.22%, 7.91%, and 13.63%, respectively.

“The reason for this result may be that nitrogen addition indirectly affects soil temperature by changing the physical properties of soil and the decomposition rate of organic matter.”Could you explain how?

Reply: Thank you for your comments. N addition may promote soil microbial activity and release heat, thereby increasing soil temperature to some extent. However, this effect is limited by a variety of factors, such as soil moisture and vegetation cover.

“In summary, it can be seen that low nitrogen concentration mainly affects soil moisture content by promoting plant growth and increasing water absorption, while high nitrogen concentration affects soil temperature by changing soil properties and organic matter decomposition rate.”But nothing is said about how and why.

Reply: Thanks for your comments. Low concentrations of nitrogen promote plant growth, increase plant absorption of water, and reduce soil moisture content. High nitrogen concentration may promote the activity of soil microorganisms, release heat, and thus increase soil temperature.

Soil moisture

Is soil moisture lower in January than in August?

Reply: Thank you for your comments. In January, the precipitation is small and the soil moisture is low, while in August, the rainy season, with high precipitation and high soil moisture.

It should be defined in the material and methods section, as well as how it is measured.

In any case I don't understand why you use Q10 here, if it is a parameter related to temperature change. Here he is analyzing the correlation between soil respiration rate and soil moisture.

Reply: Thank you for your comments. The definition and measurement methods for Q10 have been added to the paper. The addition is as follows:

Q_10_ represents the change in soil respiration rate at a temperature difference of 10 ℃, obtained by measuring the temperature difference and respiration rate before and after.

Q_10_ values from Table 1 were deleted and the description modified.

“However, Q10 values of 2.11 and 2.00 were relatively high, indicating that moisture content still affected soil respiration (p<0.05).”These values are similar to those without N

Reply: Thank you for your comments. The value was deleted.

“in suburban experimental areas was relatively low”exurb?

Reply: Thank you for your comments. The *R*^2^ values under different nitrogen application concentrations in the experimental area far away from the suburbs were lower than those in the experimental area near the suburbs. Therefore, the correlation between RS rate and soil moisture content was relatively low in experimental areas far from the suburbs.

“This is because NA can alter the nutrient conditions in the soil, thereby affecting characteristics such as soil temperature and moisture.”How does it affect soil temperature and moisture?

Reply: Thank you for your comments. N addition can affect soil acidification and degradation of soil organic matter, reduce soil water use efficiency, and lead to changes in soil moisture.

“The increase in nitrogen deposition concentration enhanced soil respiration's sensitivity to soil temperature. ”What happens with the C concentration?

Reply: Thank you for your comments. The increase in nitrogen deposition concentration enhanced soil respiration's sensitivity to soil temperature, and the enhancement of soil respiration can affect the decomposition and release of organic carbon, leading to a decrease in organic carbon content in the soil.

“3.2. Influence of NA on soil properties ”Does this section only refer to suburban areas?

Reply: Yes, this section only refer to suburban areas.

There are no significant differences between treatments, so it does not make much sense to give percentages of variation with two figures.

Reply: Thanks for your comments. This percentage change has been deleted.

“Therefore, the total nitrogen of the soil treated with low and high-nitrogen showed a slight increase compared to the total nitrogen content of the control group soil with nitrogen blank (p<0.05).”It is obvious that if there is a continuous supply of N, the content of this element in the soil increases.

Reply: Thank you for your comments. The text description has been modified. The modifications are made as follows:

It is obvious that if there was a continuous supply of nitrogen, the content of this element in the soil increased (*p>*0.05).

“3.2.3. Influence of NA on soil nitrate nitrogen”Why not just use the term “nitrate”? If you want to refer to it being expressed as N, use NO3-N

Reply: Thank you for your comments."nitrate nitrogen" has been replaced with "NO3-N". The modifications are made as follows:

3.2.3. Influence of N addition on soil NO_3_-N

Any explanation for the different behavior of the C treatment in one epoch and the other?

Reply: Thank you for your comments. C is the experimental area of high concentration of nitrogen, so in order to make the experimental effect obvious, the nitrogen added in the experimental area of C is large.

“Figure 8. Influence of NA on soil ammonium nitrogen(*Indicating p<0.05).”NH_4_-N

Reply: Thank you for your comments. The axis title of this Figure has been replaced with "NH_4_-N"

“PH”pH

Reply: Thank you for your comments. The “PH” has been replaced with "pH"

“Processing effect” What do you mean by this term?

Reply: Thank you for your comments. “Processing effect” has been deleted.

It is convenient to use the same scale in both figures.

Reply: Thank you for your comments. The scale has been modified in Fig.8.

If there are no significant differences (p>0.05) do not use **, as it is misleading.

Reply: Thank you for your comments. Figure 8 has been modified. The modification is as follows

**Figure 8.** Influence of N addition on soil heterotrophic respiration.(*Indicating *p<*0.05).

“In the suburban experimental area, when the concentration of nitrogen deposition was A, the Respiration Soil (RS), Respiration Autotrophic (RA), and RH were 0.621, 0.795, and 0.559, respectively,”What do these values mean? It is not clear how this correlation is made?

Reply: Thanks for your comments. The meaning and correlation of these values have been added in the paper.

The RS *p*-value represents the strength of the correlation between nitrogen concentration and RS. The RA *p*-value represents the strength of the correlation between nitrogen concentration and RA. The RH *p*-value indicates the strength of the correlation between nitrogen concentration and soil relative humidity. The nitrogen concentration will affect RS, RA, and RH to obtain correlation.

“Hu J”“Li X's”

Reply: Thank you for your comments. References in the paper are quoted in the format as follows:

Hu et al.

Li et al.

“NA can improve soil moisture conditions, enhance soil water persistence, promote soil water and heat functions, enhance soil permeability, and promote soil water transport.” Has this been studied in the paper or does it refer to findings from other research? Any references?

Reply: Thank you for your comments. The content mentioned in the question is taken from the references and the appropriate references have been added as follows:

N addition can improve soil moisture conditions, enhance soil water persistence, promote soil water and heat functions, enhance soil permeability, and promote soil water transport [37].

[37]R. Hu, [L. Y.Li](https://ieeexplore.ieee.org/author/37089315392), [X. Y. Zhong](https://ieeexplore.ieee.org/author/37089953890), [N. B. Zhong](https://ieeexplore.ieee.org/author/527298357723162), [B. Wan](https://ieeexplore.ieee.org/author/37089405304), and [Q. H. Xie](https://ieeexplore.ieee.org/author/470427468896055), "Spatial Distribution Information of Temperature-Humidity-Strain of Soil Cultural Relics During Salt Damage Process," IEEE Sensors Journal, vol. 23, no. 24, pp. 30403-30412, Dec, 2023, https://doi.org/ 10.1109/JSEN.2023.3328017.

There are few references to other works in the discussion.

Reply: Thank you for your comments. More contrasts have been added in the discussion section. The addition is as follows:

The adjustment of microbial community structure, the change of enzyme activity, and the growth of plant roots all require a certain time period in response to the increase of external nitrogen, so significant changes in respiration rate may not be observed in the short term. This result is similar to Yang et al.'s study on RS and temperature sensitivity to N Addition rate, both of which indicate that the short-term effect of N addition on RS is not significant[34]. The seasonal changes had a significant impact on RS, indicating that seasonal factors such as temperature and humidity play a more significant role in regulating RS. Although the interaction of N addition did have an impact on RS, the impact of N addition was relatively small compared to the impact of seasonal changes on RS (*p<*0.05). The above results are consistent with Li et al.'s study on the seasonal changes in RS caused by water N Addition in temperate grasslands over four years[35].

The references:

[34] Y. Yang, T. Li ,P. Pokharel, L. Liu, J. Qiao,Y Wang, & S. X. Chang. “Global effects on soil respiration and its temperature sensitivity depend on nitrogen addition rate,” Soil Biology and Biochemistry, vol.174, no. 3, pp. 108814-108820, October2022, <https://doi.org/10.1016/j.soilbio.2022.108814>

[35]Z. L. Li, Q. Peng , Y. S. Dong , Y. He, Z. Yan, & Y. Guo. “Response of soil respiration to water and nitrogen addition and its influencing factors: A four-year field experiment in a temperate steppe,” Plant and Soil, vol.471, no. 5, pp. 1-16, = December 2021, https://doi.org/10.1007/s11104-021-05221-3

“At the same time, changes in soil properties and decomposition rate of organic matter caused by nitrogen addition also indirectly affect soil temperature, which in turn affects soil respiration. ”

Has the decomposition of organic matter been measured?

Reply: Thanks for your comment. This paper did not measure the decomposition of organic matter, so the content of the paper has been adjusted. The content after adjustment is as follows:

At the same time, changes in soil properties caused by N addition also indirectly affect soil temperature, which in turn affects RS.

Write the references according to the journal rules.

Reply: Thank you for your feedback. The reference format has been adjusted according to the Journal guide.
